# Evidence that RXFP4 is located in enterochromaffin cells and can regulate production and release of serotonin

**DOI:** 10.1042/BSR20221956

**Published:** 2023-04-06

**Authors:** Shalinda J.A. Fernando, Qian Wang, Debbie L. Hay, Ross A.D. Bathgate, Peter R. Shepherd, Kate L. Lee

**Affiliations:** 1 Faculty of Medical and Health Sciences, University of Auckland, Auckland, New Zealand; 2 Maurice Wilkins Centre for Molecular Biodiscovery, New Zealand; 3 Department of Pharmacology and Toxicology, University of Otago, Dunedin, New Zealand; 4 Florey Institute of Neuroscience and Mental Health and Department of Biochemistry and Pharmacology, University of Melbourne, Parkville, Victoria, Australia

**Keywords:** Enterochromaffin, Gut motility, INSL5, RXFP4, Serotonin

## Abstract

RXFP4 is a G protein–coupled receptor (GPCR) in the relaxin family. It has recently been recognised that this receptor and its cognate ligand INSL5 may have a role in the regulation of food intake, gut motility, and other functions relevant to metabolic health and disease. Recent data from reporter-mice showed co-location of Rxfp4 and serotonin (5-HT) in the lower gut. We used human single-cell RNA sequence data (scRNASeq) to show that RXFP4 is in a subset of gut enterochromaffin cells that produce 5-HT in humans. We also used RNAScope to show co-location of Rxfp4 mRNA and 5-HT in mouse colon, confirming prior findings. To understand whether RXFP4 might regulate serotonin production, we developed a cell model using Colo320, a human gut-derived immortalised cell line that produces and releases serotonin. Overexpression of RXFP4 in these cells resulted in a constitutive decrease in cAMP levels in both the basal state and in cells treated with forskolin. Treatment of cells with two RXFP4 agonists, INSL5 derived peptide INSL5-A13 and small molecule compound-4, further reduced cAMP levels. This was paralleled by a reduction in expression of mRNA for TPH1, the enzyme controlling the rate limiting step in the production of serotonin. Overexpression of RXFP4 also attenuated the cAMP-induced release of serotonin from Colo320 cells. Together this demonstrates that serotonin producing enterochromaffin cells are the major site of RXFP4 expression in the gut and that RXFP4 can have inhibitory functional impacts on cAMP production as well as TPH1 expression and serotonin release.

## Introduction

The human relaxin family peptides include relaxin-1, relaxin-2 and relaxin-3 as well as insulin-like peptides (INSL3, INSL4, INSL5 and INSL6) all share structural features with the other members of the insulin superfamily peptides; insulin, insulin-like growth factor-1 and 2 (IGF-1 and IGF-2) [1,2]. The relaxin family peptides bind to and activate a group of GPCRs—relaxin family peptide receptors RXFP1–4. INSL5 binds to RXFP4 (also known as GPCR142 and GPR100) with high affinity. Both RXFP4 and RXFP3, the receptor for relaxin-3, couple to G_αi_ proteins and therefore inhibit adenylyl cyclase to suppress cAMP [3]. Although INSL5 expression is restricted to a subset of L-cells of the distal colon and rectum, RXFP4 expression is potentially located in several tissues including brain and kidney according to the Human Protein Atlas (proteinatlas.org) but is by far most abundant in the GI tract and especially the colon [3–6]. Detection of GPCRs, particularly those of low abundance is technically challenging, however the use of reporter mouse lines has recently added further evidence for RXFP4 expression in the CNS [7]. Until recently, RXFP4 in the gut was thought to be specifically expressed in the enteric neurons [8,9].

Understanding the biological roles of INSL5 and RXFP4 has been hindered by conflicting data between INSL5-/- and RXFP4-/- mice as well as studies in individual tissue models. INSL5 levels are responsive to fasting [5], microbiotic metabolism [10], and altered glucose homeostasis is a key feature of the INSL5-/- mouse [4]. Therefore, the role of INSL5 in metabolic health has been of great interest. The RXFP4-/- mouse has altered food intake, in particular lower intake of high fat diet, further supporting an orexigenic role of this ligand/receptor pair [5]. However, as INSL5 is co-secreted with GLP-1 and PYY, the latter being anorexigenic, results from physiological studies have yet to make clear the overall effect of these hormones on food intake [11]. INSL5 has also been suggested as being important for pancreatic β-cell function but findings to date are conflicting [12,13].

One role for which there is clear functional evidence for this ligand pair is in regulating gut motility [8,9]. INSL5 analogue (INSL5-A13) was able to accelerate bead propulsion dose-dependently, in mice treated with loperamide to induce constipation, but this effect was not seen in RXFP4 -/- mice [9]. These findings are further validated by use of a novel antagonist of RXFP4 which was able to block agonist-induced acceleration of colon motility in mice [8]. This has opened an interesting area of research as RXFP4-INSL5 could be a potential treatment target for constipation and other colon motility disorders. This has sparked interest in understanding the mechanism by which RXFP4 achieves these effects. Therefore, we sought to further explore the cell-type specificity of RXFP4. A recent publication commented that *Rxfp4* was expressed in colonic *Tph1* positive cells [11] in their mouse single cell RNASeq dataset [14] although details were not provided. In addition, recently published histological analysis of thick sections from the colon, distal colon and rectum of Rfxp4-reporter mice have revealed a large proportion (60–90%) of co-expression of Rxfp4 and 5-HT, and using super-resolution microscopy, 5-HT expressing cells were also found to be in proximity ‘intertwined’ with oxyntamodulin/PYY expressing L-cells [15,16]. Here we use scRNAseq datasets to show, for the first time, that RXFP4 colocalises with enterochromaffin cells (EC) in humans. We also use RNAScope combined with immunohistochemistry to show that RXFP4 and serotonin co-localise in mouse gut cells confirming previous findings using an alternative experimental technique. In addition, we used a cell model to study RXFP4 signalling *in vitro* by modulating RXFP4 levels in a human colorectal adenocarcinoma cell line (Colo320) that has been shown to express TPH1 and secrete 5-HT. This provides the first direct evidence that RXFP4 can modulate 5-HT production and secretion.

## Methods

### Materials

INSL5-A13, a shortened analogue of human INSL5 was synthesised as previously described [17]. Compound 4 was synthesised by SYNthesis Med Chem (Parkville, Australia). The activity of this compound was tested in Chinese Hamster Ovary (CHO) cells expressing human RXFP4 and was demonstrated to be similar to the published activity [18]. Other chemicals used were forskolin (Abcam, U.K. #ab120058) 3-isobutyl-1-methylxanthine (IBMX, Sigma Aldrich, U.S.A. #I5879) and pertussis toxin (Abcam #ab124299). Antibodies and primers are shown in Table 1.

**Table 1:** Antibodies and primers

Species	Target	Orientation	Nucleotide sequence 5′-3′
**Human**	RXFP4	Forward	ACCAATCTCTGATGCCCTGC
		Reverse	TCGGAGCATCATCAGCACTC
	RXFP4 insert	Forward	TCCGCCCCATTGACGCAAA
		Reverse	TACCCACAGCACCGCCAA
	TPH1	Forward	CGTCCTGTGGCTGGTTACTTA
		Reverse	TTCATGGCAGGTATCTGGCTC
	HPRT	Forward	AGTTTGTTGTAGGATATGCCCTTGAC
		Reverse	CTCTCATCTTAGGCTTTGTATTTTGCT
	**β**-Actin	Forward	CATTGCCGACAGGATGCA
		Reverse	CTCAGGAGGAGCAATGATCTTGA

Antibody	Supplier	Catalogue	Dilution
**Serotonin**	Abcam	ab6336	1:2000
**GFP**	Invitrogen	MA5-15256	1:2000
**β-Actin**	Sigma	A1978	1:20,000

### RNASeq data mining

The Bbrowser database (version 2.6.21) was searched for datasets relevant to human GI tract using key words ‘colon’, ‘gut’, ‘enteroendocrine’, ‘RXFP4’, and ‘INSL5’. Three datasets which included an enteroendocrine (EEC) cell cluster were downloaded for analysis (dataset #1 [19], dataset #2 [20], dataset#3 [21]). Each downloaded dataset was analysed separately. Expression levels of *RXFP4, INSL5* and other genes were queried. Specifically, the EEC cluster was selected and gene expression of the cells within the cluster was queried to generate a heat map to reveal co-expression of RXFP4 and INSL5 with markers of L-cells and EC cells. Finally, *RXFP4*-expressing EECs were selected, and marker genes enriched in these cells compared with all the other cells in the dataset were queried.

### Animals and tissue preparation

Animal studies took place at the University of Auckland. Tissues were collected from untreated mice being euthanised by CO_2_ for an unrelated study approved by the University of Auckland Animal Ethics Committee (AEC002174 and AEC001435) and thus animals were treated in accordance with the NZ Animal Welfare Act (1999) and the Guide for the Care and Use of Laboratory Animals and complied with the ARRIVE guidelines. The dissected colons from 14- to 16-week-old male CD1 mice or 4- to 7-month-old Rxfp4-/- mice [5] were flushed with PBS to remove all faecal pellets from the colon. The colons were cut open along the longitudinal axis and lay flat with mucosa facing upwards. Starting from the proximal end, each stretched colon was rolled around a needle to form a ‘colon Swiss-roll’. The colon Swiss-rolls were placed in an O.C.T. filled cryomold. The O.C.T. was allowed to solidify on dry ice and stored at −80°C. Ābout 8–10 µm thick sections were cut using a cryostat microtome (Leica, #CM3050 S) and mounted on SuperFrost plus slides (Bio Strategy, #EPPM4951PLUS). Each slide contained 4–8 consecutive sections. The slides were allowed to dry at −20°C for 1 h before storing at −80°C.

### Histology and RNAScope

The slides with fresh frozen tissue sections or 8-well chamber slides (Corning, #354118) containing 5 × 10^5^ Colo320 cells were immersed in pre-chilled 4% PFA and fixed for 15 min at 4°C and dehydrated in increasing concentrations of ethanol. The fixed tissue sections and Colo320 cells were then pre-treated with RNAScope® protease IV and protease III (ACD Bio, #ADV322340) respectively. RXFP4 mRNA expression in different tissues and cells were identified using RNAScope® Fluorescent multiplex assay (ACDbio, #ADV320851). The mouse RXFP4 and control probes (#ADV317581, *DapB* #ADV310043, Mm *Polr2a* #ADV312471) were purchased from ACD Bio. Immunohistochemistry (IHC) staining was performed immediately after RNAScope® assay on the same tissue sections by blocking in 2% horse serum and 5% BSA followed by primary antibody incubation (anti-serotonin, Abcam #6336) overnight at 4°C. Secondary antibody incubation (Invitrogen #A31572) was followed by mounting with Prolong diamond antifade mountant with DAPI (Life technologies #P36962). RNAScope and IHC stained slides were visualised using Zeiss Axio Imager M2 fluorescence microscope (Carl Zeiss AG, Germany) fitted with a Photometrics Prime sCMOS monochrome and Olympus DP72 CCD colour cameras. The fluorescence filter sets included DAPI, FITC (green), Texas red (red), and Cy5 (infra-red) filters.

### RXFP4 overexpression in Colo320 cells

Lentiviral transduction-mediated overexpression of RXFP4 in Colo320 cells packaged in Lenti-X™ 293T cells (Mediray #CLT632180) by combining *pLenti X1 EF1a 1xHA RXFP4 IRES GFP* (Sequence file available at figshare.com; 10.6084/m9.figshare.21084898) with *pMDLg-pRRE, pCMV-VSV-G* and *PRSV-Rev* and transfecting with Lipofectamine 2000. Colo320 cells were then infected with 1 ml of viral particles (1 × 10^−6^ titre) for 24 h followed by cell sorting for GFP positive cells after 48 h. Individual clones were expanded to check final RXFP4 gene expression levels using qPCR. Transient transfection was carried out using *pLenti X1 EF1a 1xHA RXFP4 IRES GFP* expression plasmids and Lipofectamine 2000.

Colo320 cells were cultured in α-MEM (Gibco, ThermoFisher, #11995073) supplemented with 5% FBS (Sigma Aldrich #F8067) and 1% antibiotic-antimycotic solution (Gibco, ThermoFisher #15240062) in tissue culture treated T75 and T175 flasks (Corning, NY, U.S.A.) in an incubator at 37°C and 5% CO_2_. Culture medium was changed every 48–72 h. Once the cells reached 90% confluency, cells were passaged into new flasks at 1:5 ratio.

### cAMP measurement

The day prior to the experiment cells were seeded in a 96-well tissue culture treated plate (Corning, NY, U.S.A.) at a density of 20,000 cells per well. The next day culture media was replaced with 50 µl of cAMP assay media (HBSS buffer with 0.1% w/v fatty acid-free BSA, 5 mM HEPES, and 0.5 mM IBMX, pH 7.4) and incubated at 37°C for 30 min to minimise basal cAMP levels. RXFP4 agonists and forskolin (positive control) were diluted in cAMP assay media. Once the 30-min incubation was complete, 25 µl of agonists or vehicle (0.01% DMSO) were added to each well and incubated for 15 min at 37°C. Afterwards 25 µl of forskolin dilutions was added to each well and incubated for further 15 min under same conditions. Following incubation, the contents of each well were aspirated and 50 µl of ice-cold ethanol was added and plates frozen for 15 min to extract cAMP. The plates were taken out from the freezer and kept in a fume hood to allow evaporation of ethanol after which 50 µl of cAMP detection buffer (0.35% triton X-100, 50 mM HEPES, 10 mM calcium chloride, pH 7.4) was added to each well and shaken gently at room temperature for 15 min. This cell lysate was used to quantify cAMP using the LANCE™ cAMP 384 kit (PerkinElmer, U.S.A. #TRF0262).

### RNA extraction and RT-QPCR

Cells were seeded into 12-well cell culture plates (5 × 0^5^ cells per well) and cultured overnight. The cells were treated with either vehicle (0.01% DMSO) or RXFP4 agonists for 15 min and stimulated with forskolin (10 µM) and IBMX (10 µM) for different time points up to 24 h. Following treatment, cells were washed with PBS and RNA was extracted using RNeasy mini kit (Qiagen #74106) according to manufacturer’s instructions with an additional DNase step, briefly, columns were incubated with DNase l (10 µl of DNase l stock + 70 µl of buffer RDD; Qiagen #79254) for 15 min at room temperature. The concentration and quality of the eluted RNA was measured using a NanoDrop spectrophotometer (Thermo-Fisher, U.S.A.). RNA was diluted to 50 ng/µl with RNase-free water and kept on ice or stored at −80°C for future use. Reverse transcription and quantitative PCR reaction was set up using a 1-step method, Power SYBR Green RNA-to-C_T_ 1-step kit (Applied Biosystems #4389986) according to manufacturer’s instructions and performed on a QuantStudio6 PCR system. Cycle threshold (CT) values for each replicate were averaged. Relative gene expression was calculated using the Pfaffl [22]. Housekeeping genes were *Hprt* and *Actb*. Efficiencies were checked for all primer pairs and absence of amplification from genomic DNA was confirmed using reverse transcriptase-free cDNA reactions (using a 2-step kit) from our RNA preparations. Primers were designed in house and are listed in Table 1. Primer efficiencies (E) of each primer set were calculated using a standard curve of varying template RNA concentrations. Primers with efficiencies between 1.8 and 2.2 (80–120%) were deemed acceptable.

### Western blotting

Confluent cells were washed with PBS and lysed in modified RIPA buffer (150 mM NaCl, 1% Nonidet P-40, 0.5% sodium deoxycholate, 0.1% SDS, 50 mM Tris base [pH 7.4]) supplemented with protease inhibitors (5 ml of RIPA buffer supplemented with 5 µl of 1 M AEBSF, 2 µl of 10 mg/ml aprotinin, 5 µl of 1 mg/ml pepstatin A, 2 µl of 10 mg/ml leupeptin, 10 µl of 15 mM ALLN). Protein concentration determined using bicinchoninic assay (BCA) method (Pierce, USA #23224). For SDS-PAGE, 10% polyacrylamide gels were prepared in house. Protein was loaded at 20–40 µg and electrophoresis was performed in Mini-PROTEAN tetra Cell System (Bio-Rad # 1658004) at 80 V for 30 min after which the voltage was increased to 120 V until the dye front ran off the bottom of the gel. Separated proteins were transferred onto a 0.45 µm nitrocellulose membrane (Bio-Rad #1620115) using Trans-Blot Turbo transfer system (Bio-Rad) according to manufacturer’s instructions. Membranes were blocked using 1% fish gelatin in 1× TBS buffer with Tween 20. Membranes were incubated in primary antibodies listed in Table 1 at 4°C overnight. Secondary antibodies corresponding to the primary antibodies were incubated for 1 hour at room temperature (HRP-conjugated anti-rabbit IgG (1:10,000; Life technologies #31460) and HRP-conjugated anti-mouse IgG (1:20,000; Sigma-Aldrich #A4416)). Clarity ECL solution (Bio-Rad #1705061) or Western Lighting-Plus enhanced ECL solution (Perkin-Elmer #113001EA) was used and membranes were imaged using a Bio-Rad Chemidoc Imaging system. The chemiluminescent images of membranes were analysed using Image Lab software (Bio-Rad). Pixel density of each band of desired protein was normalised to the pixel density of the loading control band in the same lane.

### 5-HT assay

Cells were seeded into 12-well cell culture plates (5 × 0^5^ cells per well). The next day cells were washed and 500 µl of HBSS buffer supplemented with 0.1% BSA was added into each well. The cells were treated with either vehicle (0.01% DMSO) or INSL5-A13 for 15 min and followed by stimulation with forskolin (10 µM) and IBMX (10 µM) for 2 h. Serotonin levels secreted in cell culture supernatant from Colo320 cells were measured using a commercially available serotonin ELISA kit (Enzo Life Sciences INC, NY, U.S.A., #ADI-900-175) that has been validated by the manufacturer including for selectivity (17% cross reactivity with N-acetyl serotonin (<1% other tryptophan metabolites)). Proteins were extracted from the cells after the cell treatment and measured using BCA assay. Total protein levels were used to normalise serotonin secretion.

### Statistics

GraphPad Prism 9 was used to perform statistical analysis of the data and produce the graphs. Statistical tests used in experiments are indicated in figure legends.

## Results

### RXFP4 is expressed in 5-HT positive enterochromaffin cells

**Figure 1:**
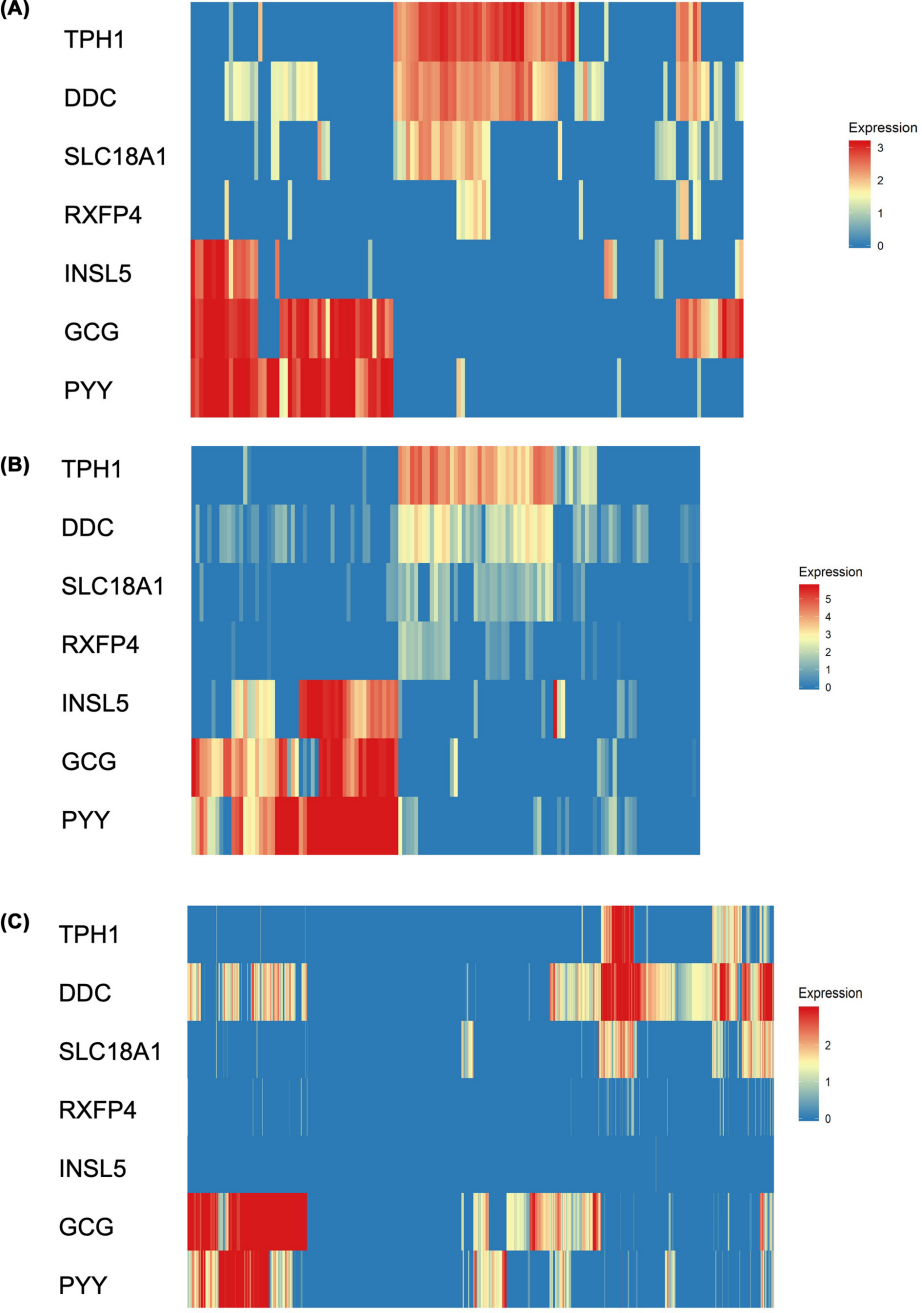
Rxfp4 is expressed in TPH1 positive enterochromaffin cells within the enteroendocrine cell clusters Heatmap of expression levels (colours indicating TPM as per individual scale bar) of enterochromaffin cell and L-cell markers including INSL5 and RFXP4 in enteroendocrine cells identified in three different RNASeq studies of digestive tract cells (**A–C**). Dataset #1, Parikh et al., 2019 (A); Dataset #2 Wang et al., 2019 (B); Dataset #3 Beumer et al., 2020 (**C**).

The RNA sequencing studies used here sampled different aspects of the human GI tract. One sampled only cells from the colon, another used cells from three regions of the GI tract and the third involving cells that were cultured and treated with NEUROG3. Human scRNASeq dataset #1 was generated from epithelial cell suspensions (>11,000 cells) from colonic biopsies of individuals with ulcerative colitis or healthy controls [19]. Dataset #2 was derived from cells taken from Ileum, colon and rectum biopsies and included data for >14,000 cells [20]. Dataset #3 comprised scRNASeq analysis of cultured organoids derived from duodenum, ileum and ascending colon that had been transduced to express NEUROG3 to push cells towards an enteroendocrine (EEC) fate [21]. Analysis of these datasets shown in Figure 1A–C reveal INSL5 to be expressed in a subset of L-cells for which glucagon (GCG) and PYY are markers. This agrees with work in mice [14]. In all three datasets RXFP4 expression was greatest in the enterochromaffin cells (EC) characterised by the expression of Tryptophan hydroxylase 1 (TPH1), DOPA decarboxylase (DDC) and SLC18A1, also known as chromaffin granule amine transporter (CGAT) or vesicular amine transporter 1 (VMAT1). There was a clear subset of these EC cells expressing RXFP4, with many EC cells containing no RXFP4. There was almost no co-expression of RXFP4 and INSL5. Bbrowser was queried to find other marker genes for RXFP4-positive enteroendocrine cells (Supplementary data file). As well as genes involved in serotonin synthesis such as TPH1, DDC and SLC18A1, other genes found to be marker genes or up-regulated in RXFP4 positive cells in more than one dataset were IGF-binding protein 3 (IGFBP3), chromogranins (CHGA and CHGB), carboxylesterase 1 (CES1) and SLC38A11. Down-regulated genes include L-cell genes GCG, PYY and INSL5 and somatostatin (SST). Tryptophan hydroxylase 1 (TPH2) was not detected in enteroendocrine cells in dataset #1 and was only present in a few cells within ‘crypt-top colonocytes’ cluster, no co-expression with RXFP4 in this group (Supplementary Figure S4). Dataset #2 had no cells in which TPH2 was detected and dataset #3 only had a few cells expressing TPH2, none of these were in the EC cell population and none co-expressed RXFP4. Substance P/Neurokinin A (TAC1), another potential marker for enteric neurons was detected in dataset #1 and 2. In dataset#1, TAC1 was not co-expressed with RXFP4 (Supplementary Figure S1). In dataset #2 there was some limited co-expression with RXFP4 (Supplementary Figure S2).

**Figure 2:**
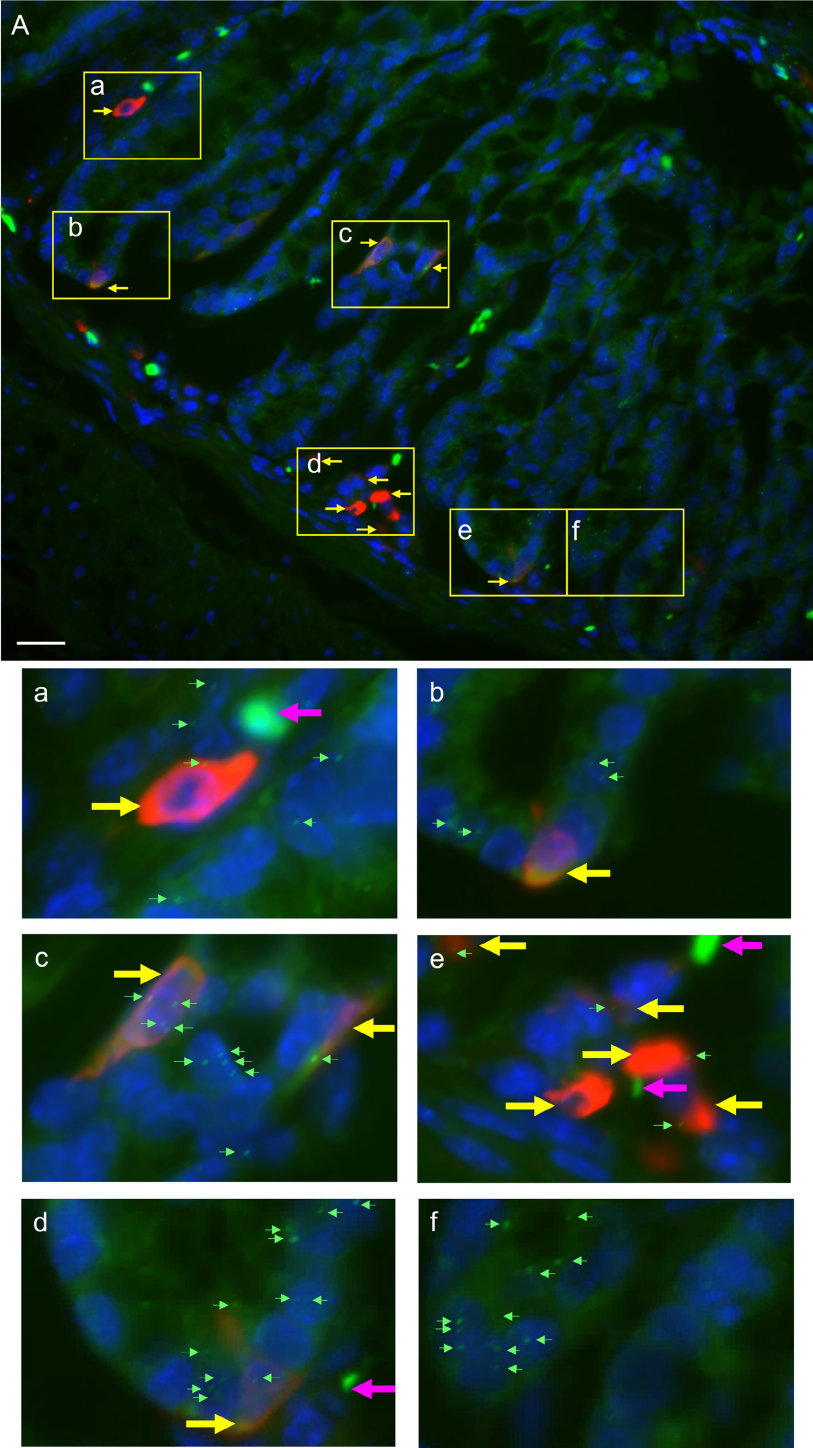
Rxfp4 within the CD1 mouse colon Rxfp4 mRNA detected by RNAScope *in situ* hybridisation using mouse Rxfp4 probe (green). 5-HT (serotonin) detected by immunohistochemistry using anti-5-HT (1:2000) antibody (red). DAPI shown in blue. Panel A shows an example image and panels (**a**–**f**) show magnified areas of tissue that contain (a–e) or do not contain (f) 5-HT positive cells (indicated by yellow arrows). Small green arrows indicate punctate RNAScope signals whereas pink arrows indicate non-specific fluorescence in the RNAScope channel which appears more diffuse. Images have been brightened to improve visibility of RNAScope signal in print. Magnification 40×, scale bar: 20 µm.

We then sought to confirm the specific localisation of Rxfp4 in serotonin producing cells in the colon using RNAScope *in situ* hybridisation combined with immunohistochemistry. Colons from male CD1 mice were used. Supplementary Figure S5 shows CD1 colons stained with positive and negative RNAScope reagents and in addition shows colons from Rxfp4-/- mice versus wild-type stained with Rxfp4 probe and co-stained with 5-HT. RNAScope signal is seen as punctate signals Figure 2a-f shows a magnified image of 5-HT positive stained cells where Rxfp4 speckles are visible. Most 5-HT positive cells have some Rxfp4 speckles, however there is Rxfp4 signal in areas not positive for 5-HT. This aligns with immunohistological analysis of a Rxfp4-reporter mouse where full quantification showed co-expression to vary across the lower GI tract, over 90% of 5-HT-positive cells were also immunoreactive for GFP [15]. These findings make it clear that INSL5-RXFP4 signalling has great potential to affect serotonin synthesis and/or secretion from EC cells.

### RXFP4 overexpression in Colo320 cells lowers cAMP response to forskolin

**Figure 3:**
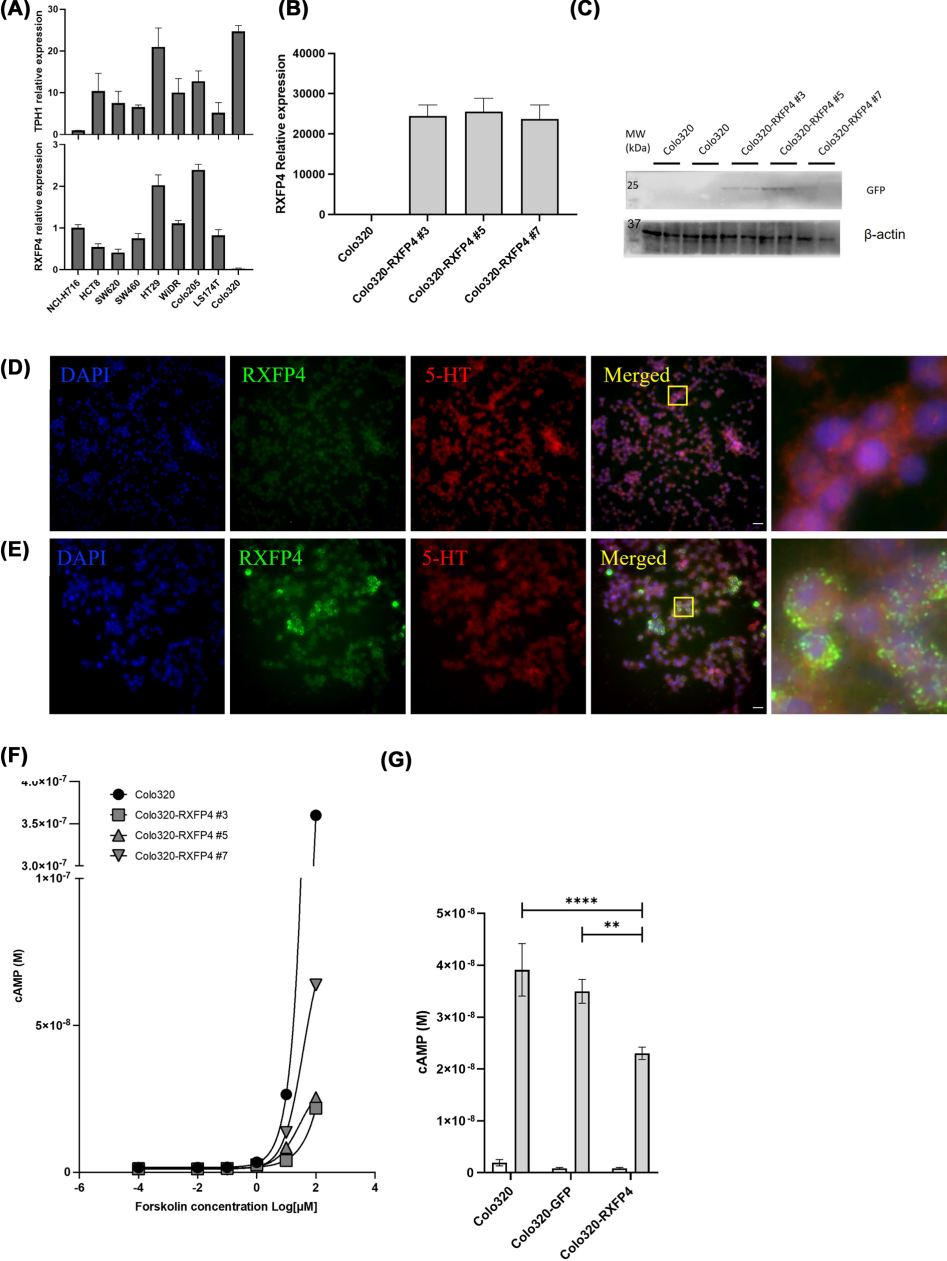
RXFP4 overexpression in Colo320 cells lowers cAMP response to forskolin TPH1 and RXFP4 mRNA expression in various human colorectal cancer cell lines relative to NCI-H716 cells measured by qPCR, *N*=4 (**A**). Baseline/untreated RXFP4 mRNA in individual clones with stable overexpression of RXFP4, *N*=3 (**B**). Western blot showing GFP levels in individual clones with stable overexpression of RXFP4, full blot image in Supplementary Figure S6 (**C**). Rxfp4 mRNA detected by RNAScope *in situ* hybridisation using mouse Rxfp4 probe (green), 5-HT detected by immunohistochemistry using anti-5-HT (1:2000) antibody (red), and DAPI shown in blue in untransfected Colo320 cells (**D**) and Colo320 cells overexpressing Rxfp4 (**E**). Forskolin stimulation of cAMP in individual clones with stable overexpression of RXFP4, *N*=1 (**F**). Forskolin (10 μM) stimulation of cAMP in Colo320 cells with transient overexpression of RXFP4 or GFP-only vector. Forskolin treatment in grey bars and vehicle control (open bars). Two-way ANOVA with Tukey’s post-hoc test comparing cell lines within treatment ****P<0.0001, **P<0.01, *N*=4 (**G**).

We set out to identify a cell line that would be suitable for studying the impact of RXFP4 on serotonin production and secretion and focussed on identifying gut derived cell lines with this characteristic. Following assessment of several human colorectal cancer cell lines for TPH1 mRNA expression we identified Colo320 cells as being suitable for use (Figure 3A). Colo320 cells have previously been shown to secrete serotonin [23,24]. As basal expression of RXFP4 is likely to be too low to see effects on cAMP in these cell lines, we chose the line with the highest TPH1 expression and transfected with a human RXFP4 expression construct. Figure 3 shows overexpression of RXFP4 mRNA (B) and overexpression of GFP protein (C) compared with parent Colo320 cells. Forskolin stimulated cAMP production was assessed in several clones of these cells and there was clear inhibition of cAMP levels in the overexpressing lines compared with parent Colo320 cells in the absence of exogenous ligand (Figure 3F). Transient overexpression of RXFP4 but not a GFP expressing control vector were able to inhibit forskolin-mediated elevation in cAMP levels indicating the suppression is specific to the presence of RXFP4 (Figure 3G).

### Inhibition of forskolin stimulated cAMP production is restored by treatment with Pertussis toxin

**Figure 4:**
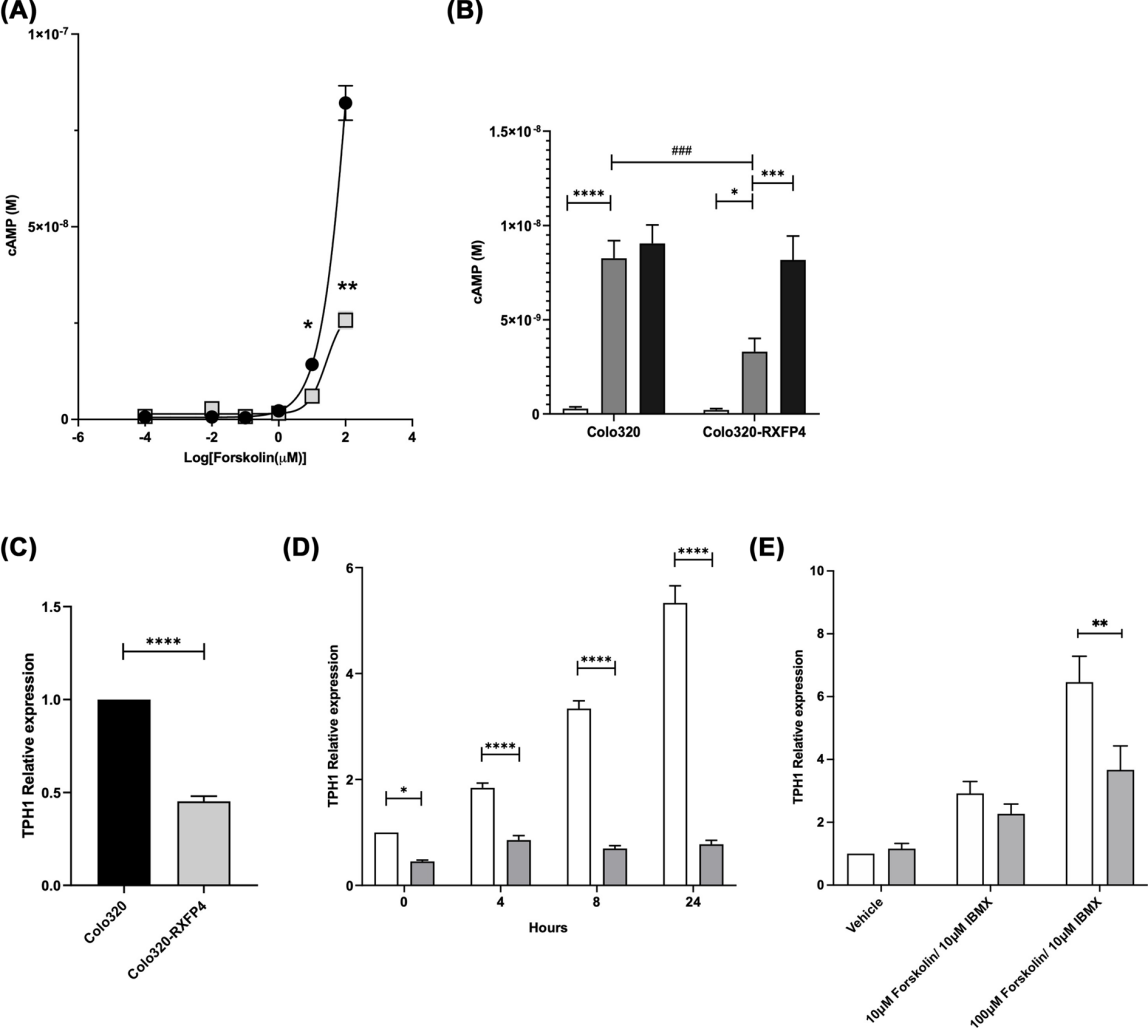
Inhibition of forskolin-stimulated cAMP production is reversed with Pertussis toxin and is associated with inhibition of TPH1 gene expression Forskolin stimulated cAMP production measure by LANCE in Colo320 clone#1 with stable over expression of RFXP4 (shaded squares) vs. un-transfected Colo320 (black circles),* N*=2. Statistics are Mixed-Effects model with posthoc Tukey’s multiple comparisons test comparing cell lines within concentrations and multiplicity adjusted p-values reported (Colo320 vs. RXFP4, *P<0.05, **P<0.01) (**A**). Forskolin stimulated cAMP production in selected Colo320 clone with stable over expression of RFXP4 with and without 100 ng/ml pertussis toxin (PTX), *N*=3. Vehicle control (open bars), 10 µm forskolin (shaded bars), 10 µm forskolin and 100 ng/ml PTX (black bars). Statistics are two-way ANOVA with Tukey’s multiple comparison test across treatments within cell line (*P<0.05, ***P<0.001, ****P<0.0001) or Tukey’s multiple tests comparing response within treatment across cell lines (###*P*<0.001). Multiplicity adjusted *P*-values reported (**B**). TPH1 mRNA levels in un-transfected and Colo320 cells with stable over expression of RXFP4, *N*=5 (**C**). TPH1 mRNA expression over time when treated with 10 µM forskolin/ 10 µM IBMX in un-transfected (open bars) and RXFP4 overexpressing (shaded bars) colo320 cells, *N*=5. Statistics are two-way ANOVA with Sidak’s multiple comparison test between cell lines (****P<0.0001) (**D**). TPH1 mRNA expression when treated with increasing concentrations of forskolin for 24 h. Un-transfected (open bars) and RXFP4 overexpressing (shaded bars) colo320 cells, *N*=3. Statistics are two-way ANOVA with Sidak’s multiple comparison test between cell lines (**P<0.01). Multiplicity adjusted *P*-values reported (**E**).

A further Colo320 clone with stable overexpression of RXFP4 was used for further evaluation, suppression of forskolin-induced cAMP for this line is shown in Figure 4A. This suppression was reversed by the addition of Pertussis toxin indicating this suppression is specific to G_αi_ activation (Figure 4B).

### RXFP4 overexpression in Colo320 cells lowers forskolin-stimulated TPH1 gene expression

TPH1 mRNA levels were found to be supressed in the RXFP4 overexpressing Colo320 clone (Figure 4C). TPH1 mRNA increases over time in Colo320 cells when treated with 10 μM forskolin and 10 μM IBMX; however, the overexpression of RXFP4 abolishes this effect (Figure 4D). TPH1 mRNA expression is increased in RXFP4 overexpressing cells when treated with 10 μM IBMX and 100 μM forskolin for 24 h, albeit at significantly lower levels than un-transfected Colo320 cells (Figure 4E).

### RXFP4 agonists can inhibit forskolin-induced cAMP, TPH1 gene expression and 5HT secretion

**Figure 5:**
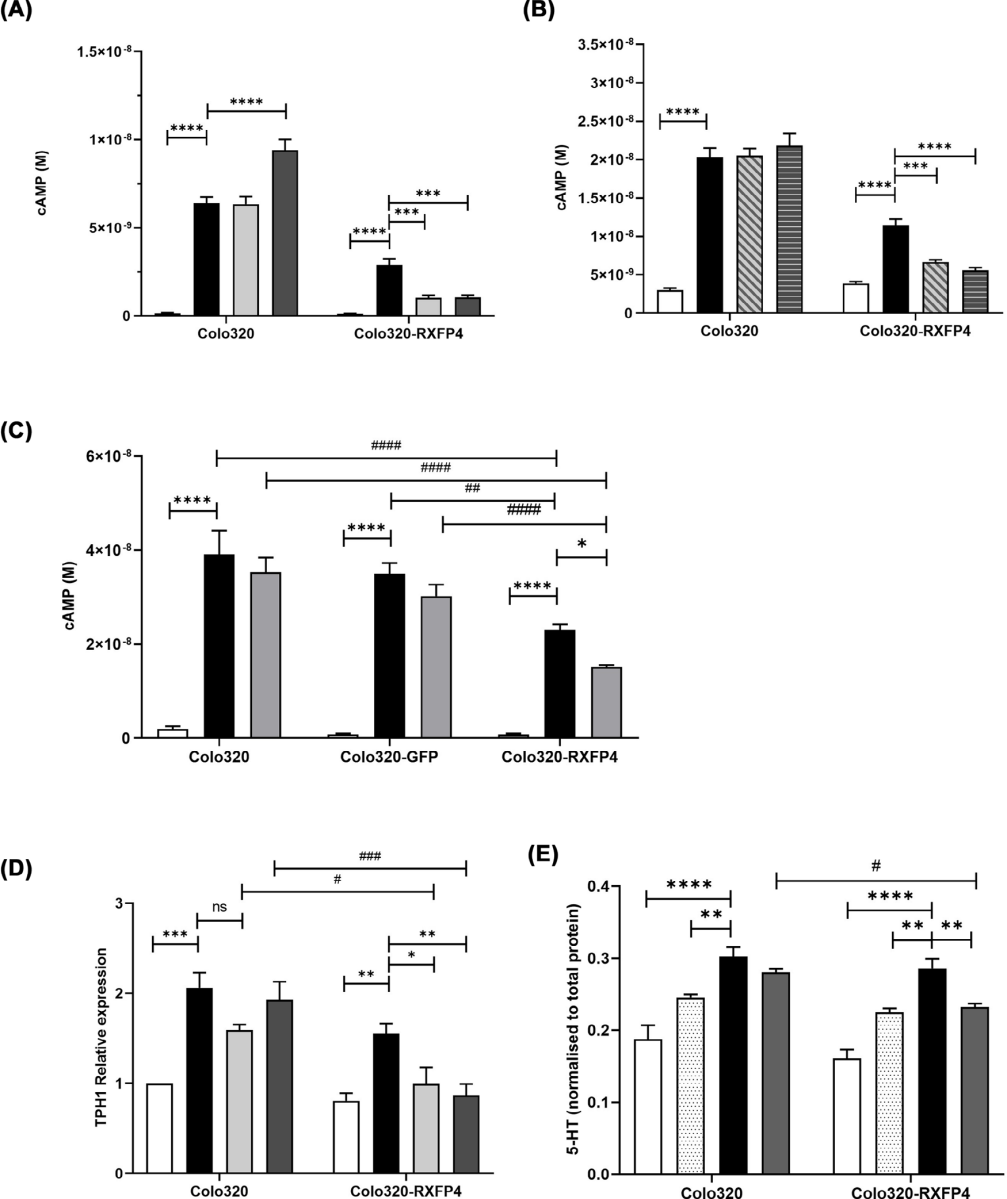
Inhibition of forskolin-induced cAMP by RXFP4 agonists is also associated with suppression of TPH1 gene expression and serotonin secretion Forskolin-stimulated cAMP production is supressed by INSL5-A13 in RXFP4 overexpressing Colo320 cells. Vehicle control (open bars), 10 µm forskolin (black bars), 10 µM forskolin and 10 nM INSL5-A13 (pale shaded bars), 10 µM forskolin and 100 nM INSL5-A13 (dark shaded bars) *N*=3. Statistics are two-way ANOVA with Dunnett’s multiple comparison test across treatments within cell line, compared with 10 µM forskolin (***P<0.001, ****P<0.0001) (**A**). Forskolin-stimulated cAMP production is supressed by Compound 4 in RXFP4 overexpressing Colo320 cells. Vehicle control (open bars), 10 µm forskolin (black bars), 10 µM forskolin and 1 nM Compound 4 (pale diagonal striped bars), 10 µM forskolin and 10 nM Compound 4 (dark horizontal striped bars), *N*=3. Statistics are two-way ANOVA with Dunnett's multiple comparison test across treatments within cell line, compared with 10 µM forskolin (***P<0.001, ****P<0.0001) (**B**). Forskolin-stimulated cAMP production is supressed by INSL5-A13 in Colo320 cells transiently overexpressing RXFP4 but not untransfected or control vector transfected cells. Vehicle control (open bars), 10 µm forskolin (black bars), 10 µM forskolin and 100 nM INSL5-A13 (shaded bars), *N*=4. Statistics are two-way ANOVA with Dunnett’s multiple comparison test across treatments within cell line, compared with 10 µM forskolin (****P<0.0001) and Tukey’s multiple comparison test across cell lines (##*P*<0.01, ####*P*<0.0001) (**C**). Forskolin-induced increase in TPH1 mRNA expression is supressed by INSL5-A13. Vehicle control (open bars), 10 µm forskolin/10 µM IBMX (black bars), 10 µM forskolin/10 µM IBMX and 10 nM INSL5-A13 (pale shaded bars), 10 µM forskolin/10 µM IBMX and 100 nM INSL5-A13 (dark shaded bars), *N* = 3. Statistics are two-way ANOVA with Dunnett’s multiple comparison test across treatments within cell line, compared with 10 µM forskolin (*P<0.05, **P<0.01, ***P<0.001) Tukey’s multiple comparison test across cell lines (#*P*<0.05, ###*P*<0.001) (**D**). Forskolin-induced increase in serotonin (5-HT) secretion is supressed by INSL5-A13. Media tested at baseline/0 hours (open bars), 4 h after addition of vehicle (hashed bars), 4 h after addition of 10 µm forskolin/10 µM IBMX (black bars), 4 h following addition of 10 µm forskolin/10 µM IBMX with 100 nM INSL5-A13 (dark shaded bars), *N*=4. Statistics are two-way ANOVA with Dunnett’s multiple comparison test across treatments within cell line, compared with 10 µM forskolin (**P<0.01, ****P<0.0001). Tukey’s multiple comparison test across cell lines (#*P*<0.05 (**E**).

Both INSL5-A13 (10 and 100 nM, Figure 5A) and compound 4 (1 and 10 nM, Figure 5B) were able to lower forskolin-induced cAMP increases in Colo320 cells stably overexpressing RXFP4. Neither agonist was able to supress forskolin-induced cAMP in the non-transfected Colo320 cells, although 100 nM of INSL5-A13 appeared to increase cAMP in these cells. Again, these experiments replicated the suppression of forskolin induced cAMP in the absence of ligand. In Colo320 cells transiently overexpressing RXFP4, 100 nM INSL5-A13 was able to supress the forskolin-induced cAMP increase. The agonist did not have this effect in the un-transfected Colo320 or Colo320 overexpressing GFP plasmid (Figure 5C). Reflecting the effect of INSL5-A13 on cAMP suppression, 10 and 100 nM INSL5-A13 was able to supress forskolin/IBMX-induced increase in TPH1 gene expression in RXFP4 Colo320 cells with no effect on un-transfected cells (Figure 5D). It is worth noting that basal levels of TPH1 mRNA in this experiment were not different in the vehicle treated cells in contrast with results in Figure 4C. Crucially 100 nM INSL5-A13 was also able to supress serotonin secretion from Colo320 cells into the cell culture medium when RXFP4 was overexpressed, but not in untransfected cells (Figure 5E).

## Discussion

While evidence from mice points to many important roles for the INSL5-RXFP4 system, in many species RXFP4 or INSL5 have become pseudogenes, particularly in carnivores [25]. While this is not the case for humans or mice it does indicate a significant amount of functional divergence is tolerated in this system and so it cannot be taken for granted that the way the INSL5-RXFP4 system functions in mice and humans will be the same. What is more there is only a moderate level of sequence conservation between mouse and human RXFP4. Therefore, it is important to independently validate cellular locations of these hormones in humans. Thus, our findings on the localisation of RXFP4 and INSL5 in humans provide important new information. We have combined several sources of information to understand RXFP4 gene expression in the population of EEC of the GI tract in humans. This analysis shows RXFP4 expression is in a specific sub-set of TPH1 positive EC cells in human colon. We also add immunohistological evidence using RNAScope to detect this GPCR revealing clear co-localisation with serotonin in mouse colon.

Even though the human scRNASeq datasets used in this study all used very different populations of GI tract cells they all reveal that a subset of serotonin secreting EC cells express *RXFP4*. Our studies do not provide insights as to exactly where in the GI tract the RXFP4 cells might be localised. However, it is interesting to note that a recent histological analysis of 5-HT positive cells in the mouse GI tract revealed close physiological contact between these cells and oxyntomodulin/PYY expressing L-cells that are the source of INSL5 which together with the data we present here supports a paracrine role in the lower GI tract for L-cell derived INSL5 [16].

Our studies identify some genes co-expressed with *RXFP4* that could potentially be part of the mechanism by which RXFP4 exerts its action. One is carboxylesterase 1 (encoded by *CES1*) which is thought of as being the liver specific carboxylesterase with CES2 being expressed in the gut. This analysis suggests CES1 may have specific expression in EC cells although its function in these cells has not been established. CES1 is the main enzyme involved in methylphenidate metabolism; a stimulant used for the treatment of attention-deficit/hyperactivity disorder [26]. Chronic administration of methylphenidate was associated subtle alterations in circulating serotonin levels [27] suggesting CES1 may serve a regulatory function in EC cell function and 5-HT release. We find IGF binding protein 3 (*IGFBP3*) is expressed in the RXFP4 positive EC cells which is a novel finding as expression of this is not thought to be abundant in gut. Loss of *IGFBP3* in mice was associated with lower levels of serotonin and dopamine in brain tissue [28] although its effects on peripheral serotonin is, as yet, unknown. However, *IGFBP3* has been implicated in regulation of gut stem cell proliferation and differentiation [29]. Interestingly, we also saw a small amount of GCG co-expression with RXFP4 which agrees with a recent immunohistological analysis of mouse gut that found a small amount of RXFP4 co-expression with Oxyntamodulin [15]. We looked at TPH2 and TAC1 to indicate the presence of enteric neuronal cell types but found little evidence for co-expression of RXFP4, although these markers were only expressed in a few cells suggesting these cell types were not sufficiently surveyed in these datasets. Although with co-expression being seen in only one or two of 1000s of cells in such studies, the potential of that signal coming from multiple cells or compromised cells cannot be excluded.

Given the known role of RXFP4 in regulating gut motility [8,9] the most important findings here relate to the potential for RXFP4 to regulate serotonin, a hormone known to play a key role in regulating gut motility [30,31]. We find *TPH1, DDC and SLC18A1* expression overlaps with *RXFP4* expression and these are crucial for the production and regulation of serotonin in EC cells [32,33]. *CHGA* and *CHGB* were also identified as marker genes in *RXFP4* positive EC cells in all three datasets. Chromogranin A and chromogranin B play important roles in regulating secretory vesicle function in neurons and endocrine cells such as of EECs [34]. Together this suggest a major function of RXFP4 may be to regulate serotonin production and/or secretion from EC cells.

Given that RXFP4 is known to be an inhibitory GPCR, our data also provides the first evidence to suggest that INSL5 binding to RXFP4 can act to negatively regulate the production and secretion of serotonin. Using our Colo320 model we provide the first evidence that overexpression of RXFP4 alone and subsequent exposure to an RXFP4 agonist both result in significant reductions in cAMP levels and *TPH1* expression. Triggering of EC secretory 5-HT granule release is achieved through the same mechanisms as other secretory cell types such as islet β-cells; via Ca^2+^/cAMP [35,36]. This is consistent with other findings in other cell types that show that cAMP is involved in up-regulating TPH1 expression [37]

In summary, we find that RXFP4 is localised to a subset of EC cells in humans and confirm co-expression of 5-HT and RXFP4 in mouse colon. In addition, we show that INSL5-RXFP4 signalling has the ability to oppose serotonin production and release. This seems paradoxical as there is strong evidence that serotonin promotes anterograde gut motility [38] and agonists of RXFP4 also increase gut motility, at least as shown by colonic bead expulsion assays [9]. Further, a 5-HT3 receptor antagonist Alosteron was recently shown to block INSL5-A13 action on gut motility in the bead expulsion assay [15] However, given that INSL5 is produced in cells in the distal colon [5] and that we find RXFP4 is only found in a subset of EC cells we postulate that the INSL5-RXFP4 system may be having a specific effect on the retrograde propulsion waves that are generated in this part of the colon [39]. Interestingly these are shown to increase after a meal, as does production of INSL5. Thus, INSL5 mediated reduction in serotonin production may contribute to colonic bead expulsion by reducing the intensity of these retrograde pulses.

Future work will need to focus on the extent to which RXFP4 signalling impacts serotonin and whether RXFP4 signalling acting via serotonin regulation or otherwise in the gut has impacts relevant to human health and disease.

## Supplementary Material

Supplementary Figures S1-S6 and Supplementary Data File

## Data Availability

Single cell datasets were not generated nor owned by the authors and are available online. All other relevant data is provided within the manuscript, figures and supplementary data. Other data used and/or analyzed during the present study are available from the corresponding author on reasonable request.

## Open Access

Open access for this article was enabled by the participation of The University of Auckland in an all-inclusive *Read & Publish* agreement with Portland Press and the Biochemical Society under a transformative agreement with CAUL.
