# Supplementary material for: Evidence that RXFP4 is located in enterochromaffin cells and can regulate production and release of serotonin
Source: Biosci Rep. 2023 Apr 6;43(4):BSR20221956. doi: 10.1042/BSR20221956 (PMC10086114; doi:10.1042/BSR20221956)
Supplement: Supplementary Figures S1-S6 and Supplementary Data File [file BSR-2022-1956_supp.pdf]

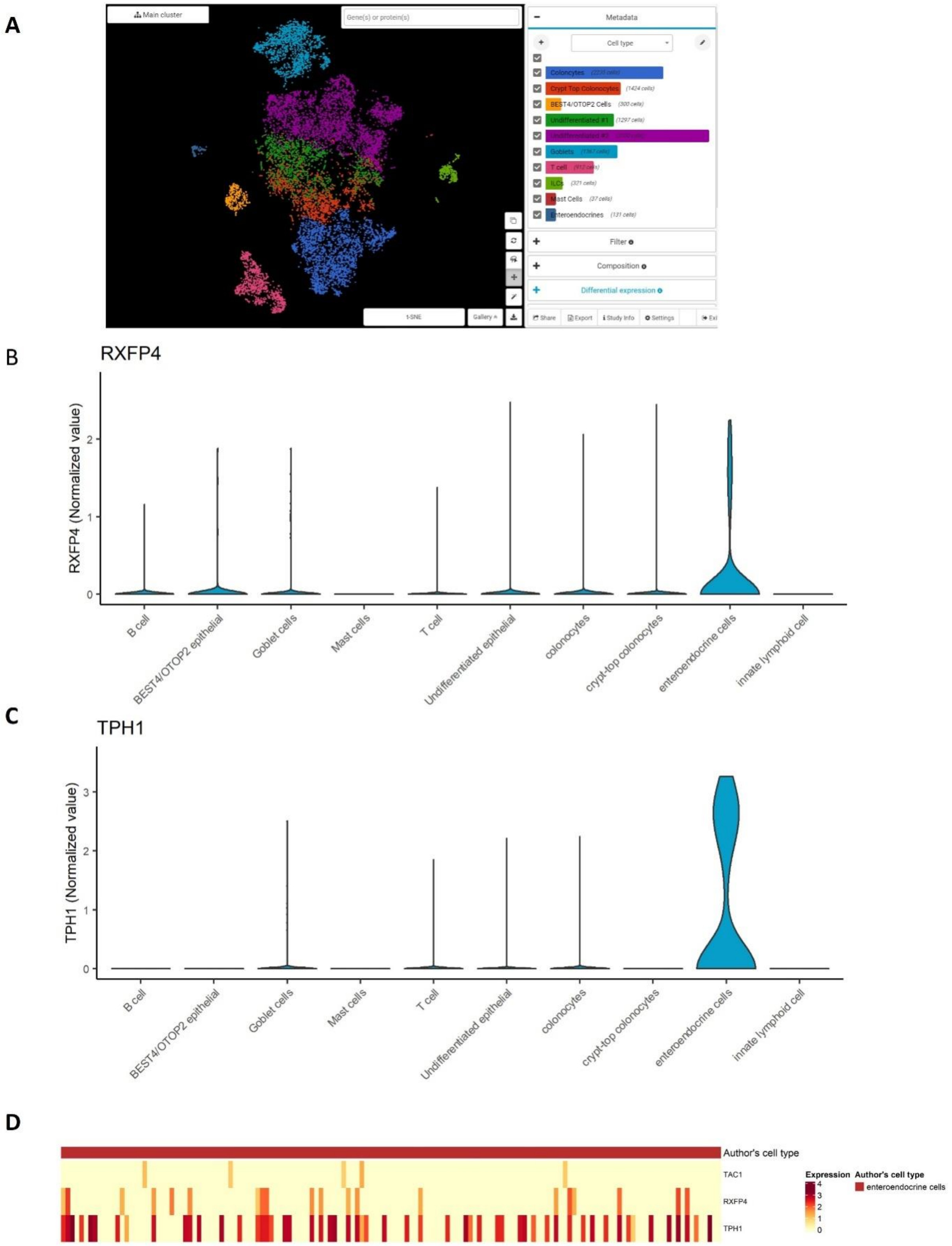

Suppl Figure 1:tSNE plot for dataset #1 (A) showing RXFP4 is almost exclusively expressed in EEC cells (B), TPH1 is also almost exclusively expressed in the EEC cluster (C). Showing expression of TAC1 (gene for precursor of substance P) only expressed in a small number of EEC cells and not co-expressed with RXFP4 (D) Plots generated by Bbrowser.

A

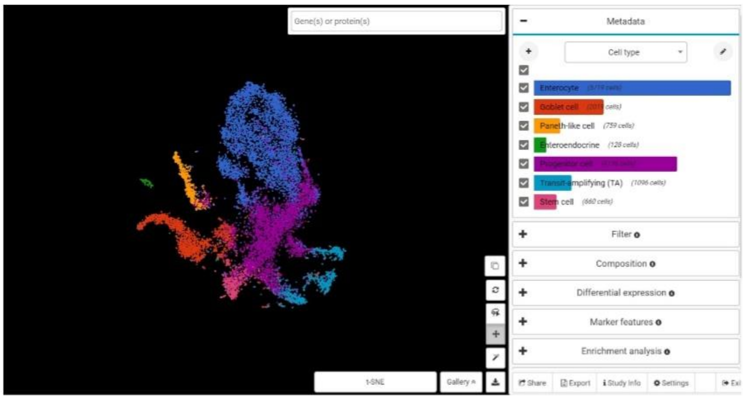

B

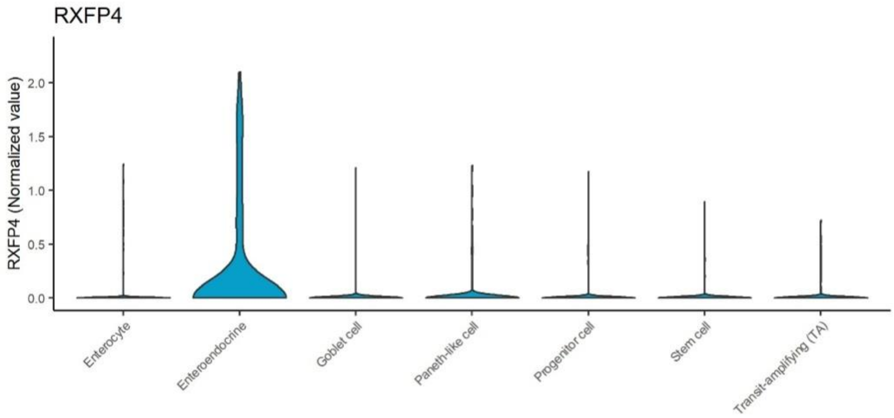

C

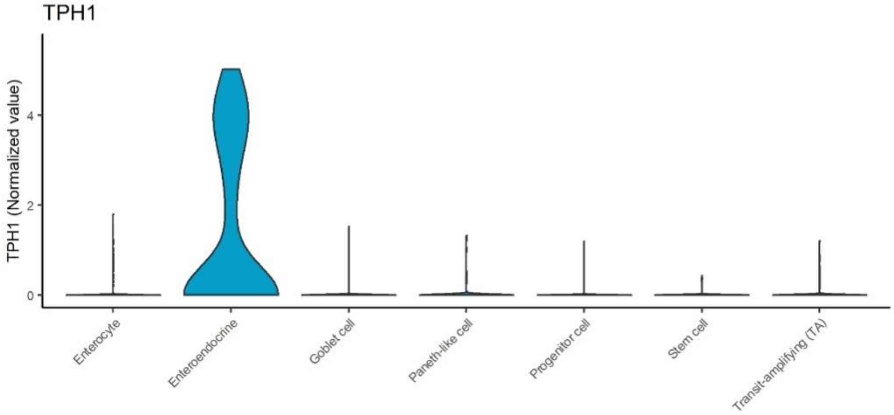

D

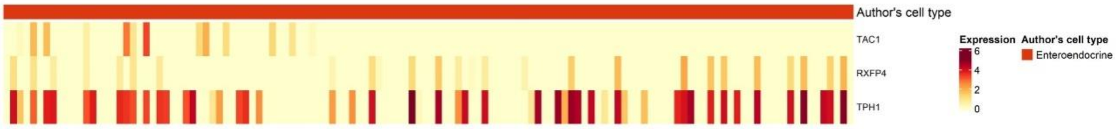

Suppl Figure 2:tSNE plot for dataset #2 (A) showing RXFP4 is almost exclusively expressed in EEC cells (B), TPH1 is also almost exclusively expressed in the EEC cluster (C). Showing expression of TAC1 (gene for precursor of substance P) only expressed in a small number of EEC cells and found co-expressed with RXFP4 in a single cell (D) Plots generated by Bbrowser.

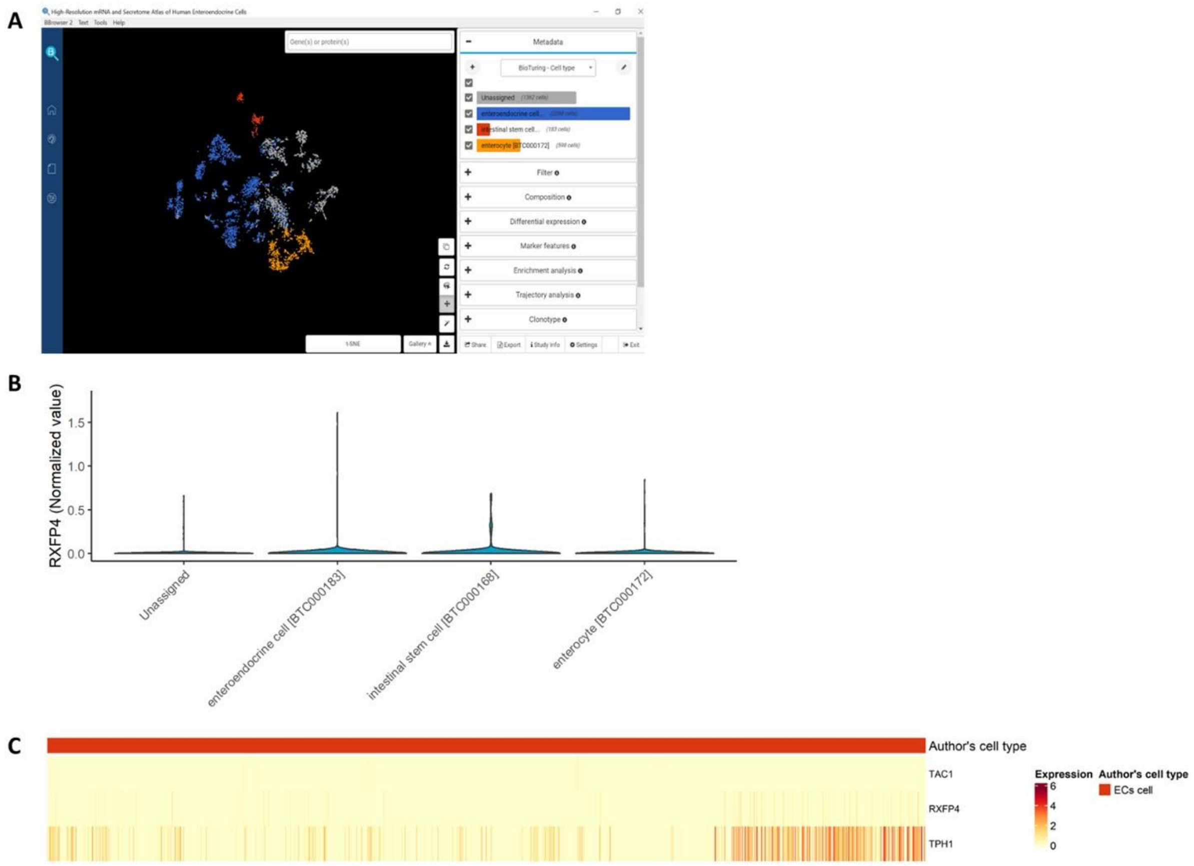

Suppl Figure 3:tSNE plot for dataset #3 (A) showing RXFP4 is most expressed in EEC cells (B). Showing expression of TAC1 (gene for precursor of substance P) only expressed in a small number of EEC cells (C) Plots generated by Bbrowser.

A

B

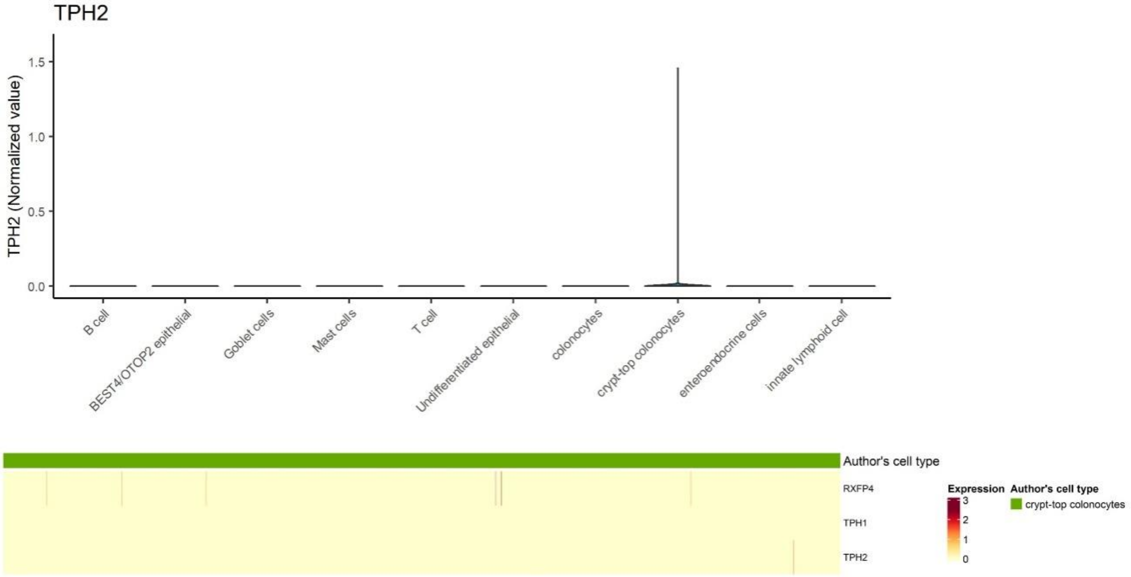

Suppl Figure 4: Expression of TPH2 in Dataset 1 and 3. (A) TPH2 was detected in 'crypt-top colonocytes' cell group only in dataset #1. (B) In crypt-top colonocytes of dataset #1 there was no co-expression with RXFP4 which was also detected in a few cells in this group.

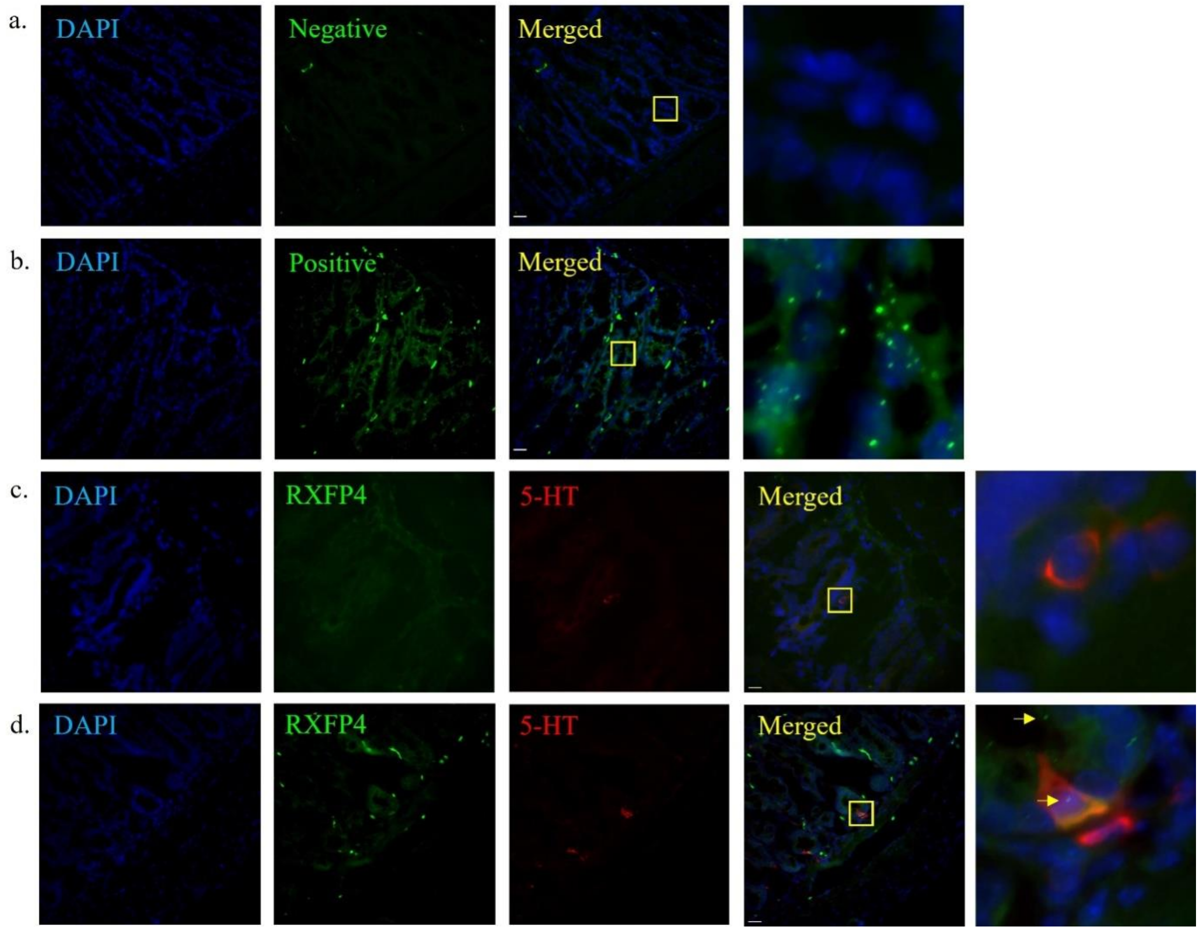

Suppl Figure 5: Optmisation of RNAScope assay. CD1 mouse colon tissuestained with RNAScope negative probe (a), RNAScope positive control probe (b) and colons from Rxfp4 -/- (c) and wild-type controls (d). *Rxfp4* mRNA detected by RNAScope*in situ* hybridisation using mouse Rxfp4 probe. 5-HT detected by immunohistochemistry using anti-5-HT (1:2000) antibody.

Full image

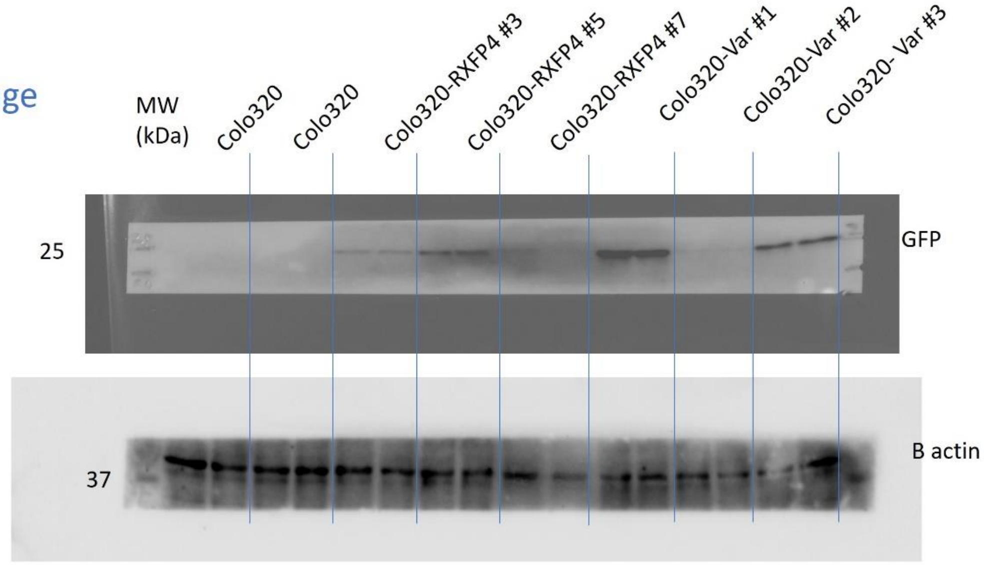

Figure includes relevant clones (RXFP4# 3, #5 and #7) only.

Suppl Figure 6: Full western blot image for figure 3C.

Suppl Table 1: Genes significantly up- or down-regulated in *RXFP4*-positive EECs vs non-RXFP4 EEC cells in three scRNASeq datasets analysed (#1 left, #2 middle, #3 right). Data shown;gene name, % of cells positive for expression in group 1 (RXFP4 expressing EECs), % of cells positive for expression in group 2 (non-RXFP4 expressing EECs), Log2FC (between RXFP4-expressing and non RXFP4 expressing EECs) and p-value indicating whether difference is significant).

Dataset	Gene Name	% group 1	% group 2	Log2FC	P-value
#1	RXFP4	100	0	1.131	6.80E-15
#1	TPH1	85	30	1.102	0.00003

Dataset	Gene Name	% group 1	% group 2	Log2FC	P-value
#2	LCN15	31	10	2.812	0.0468
#2	TPH1	88	28	2.324	6.67E-08
#2	CHGA	96	73	2.141	9.25E-06
#2	IGFBP3	81	18	1.881	2.03E-06
#2	CHGB	92	58	1.677	4.42E-06
#2	CES1	54	19	1.617	0.00199
#2	RGS2	85	39	1.613	2.52E-06
#2	DDC	100	56	1.537	2.05E-08
#2	SYT13	92	53	1.447	3.30E-08
#2	PCSK1	85	40	1.432	0.00001
#2	ID3	77	55	1.391	0.02806
#2	RXFP4	100	0	1.366	0.00001
#2	GCLC	85	43	1.129	0.00012
#2	SLC38A11	77	28	1.11	3.31E-06
#2	SLC18A1	85	25	1.077	4.30E-08
#2	TTR	85	53	1.049	0.01204
#2	SLPI	19	40	-1.014	0.01535
#2	HLA-DRA	23	47	-1.048	0.01535
#2	UCN3	27	55	-1.065	0.00593
#2	LYPD8	15	29	-1.226	0.0451
#2	SPINK4	38	57	-1.633	0.03839
#2	GUCA2A	46	52	-1.915	0.03728
#2	GCG	8	53	-2.902	0.00011
#2	PYY	35	58	-3.531	0.00021
#2	INSL5	15	43	-4.168	0.00705
#2	NTS	19	18	-6.942	0.01464

Dataset	Gene Name	% group 1	% group 2	Log2FC	P-value
#3	CHGB	97	36	4.149	5.73E-32
#3	LGALS4	98	38	3.43	6.23E-25
#3	CHGA	100	84	3.335	1.18E-29
#3	REG4	98	54	3.219	2.85E-26
#3	SCG5	98	24	3.18	2.14E-33
#3	OR51E1	98	20	3.166	3.04E-25
#3	CES1	95	22	3.141	4.93E-29
#3	PSCA	85	19	3.13	9.53E-23
#3	C9ORF16	100	29	3.126	3.8E-32
#3	TPH1	88	21	3.112	8.41E-23
#3	NEUROD1	93	33	3.078	2.02E-26
#3	DDC	100	36	3.041	1.41E-34
#3	FCGBP	85	14	2.964	2.19E-22
#3	S100A6	100	53	2.958	1.33E-28
#3	TMSB4X	100	39	2.95	5.79E-29
#3	KCTD12	98	39	2.847	2.48E-29
#3	PAM	90	27	2.809	3.93E-19
#3	TFF3	98	37	2.777	5.99E-28
#3	TFF1	86	25	2.776	1.93E-14
#3	FOS	97	32	2.765	4.44E-28
#3	SYT13	98	29	2.761	1.2E-29
#3	IER2	93	24	2.727	4.24E-24
#3	CDKN1A	93	28	2.696	1.31E-17
#3	COX5A	97	28	2.689	6.37E-28
#3	RGS2	95	28	2.686	5.4E-28
#3	CACNA2D1	92	22	2.676	3.71E-28
#3	LINC00261	78	20	2.668	6.84E-18
#3	MS4A8	69	15	2.654	1.39E-18
#3	FEV	100	41	2.647	8.81E-30
#3	GSTK1	98	25	2.646	1.18E-31
#3	JUNB	97	27	2.644	2.08E-26
#3	B2M	98	45	2.637	4.03E-27
#3	SLC29A4	98	32	2.617	8.31E-29
#3	COX6A1	97	35	2.613	1.71E-24
#3	RPS12	98	41	2.608	4.33E-28
#3	GAPDH	98	46	2.605	3.52E-28
#3	CLDN7	93	28	2.602	1.5E-26
#3	RPS3	98	35	2.59	1.02E-29
#3	SLC18A1	97	23	2.587	1.38E-28
#3	INSM1	97	35	2.586	9.77E-27
#3	AGR2	95	27	2.58	1.02E-24
#3	CRYBA2	97	26	2.576	3.43E-27
#3	EEF1A1	100	54	2.575	4.24E-27
#3	ACTG1	98	39	2.551	8.2E-27
#3	KRT20	85	21	2.533	2.78E-20
#3	EGR1	92	28	2.528	1.78E-23
#3	GNAS	98	53	2.527	1.17E-27
#3	RPL41	100	53	2.522	1.08E-28
#3	ADH4	75	15	2.515	2.84E-12
#3	TPT1	98	39	2.51	6.8E-27
#3	SLC25A5	92	24	2.501	1.2E-24
#3	PCSK1N	98	40	2.5	8.92E-29
#3	RPLP1	98	39	2.491	1.59E-29
#3	CPE	98	27	2.474	2.09E-24
#3	RPS3A	98	39	2.467	3.07E-26
#3	RPS2	98	35	2.465	1.65E-29
#3	TSPAN1	92	28	2.461	4.35E-25
#3	KRT8	100	44	2.449	8.96E-28
#3	SCD	95	28	2.444	7.04E-22
#3	FTL	95	33	2.441	1.53E-23
#3	HMGCS1	92	22	2.432	1.24E-24
#3	RPL15	98	41	2.423	1.08E-25
#3	RPL13	100	38	2.421	1.29E-28
#3	SCGN	98	36	2.421	1.05E-29
#3	HSPA8	100	33	2.416	5.89E-29
#3	IGFBP3	97	27	2.416	5.71E-26
#3	RPS11	98	39	2.415	3.66E-29
#3	PEBP1	98	32	2.411	2.97E-26
#3	RAB3B	97	28	2.409	6.2E-25
#3	VGF	83	16	2.385	1.03E-21
#3	UBB	100	34	2.382	8.02E-29
#3	EEF1G	98	33	2.376	1.78E-29
#3	RPS27	98	42	2.37	3.44E-28
#3	RASA4	97	27	2.369	1.48E-25
#3	NEAT1	97	34	2.367	5.46E-23
#3	REEP5	95	24	2.35	1.62E-25
#3	RPS29	97	36	2.342	4.15E-29
#3	RPL37A	98	34	2.339	1.22E-28
#3	TAGLN2	97	27	2.337	1.29E-28
#3	CTSC	90	20	2.337	1.64E-22
#3	CTSB	88	22	2.331	3.67E-19
#3	GCH1	100	32	2.326	2.6E-27
#3	RPS8	98	36	2.321	1.72E-26
#3	ENO1	98	30	2.314	1.87E-27
#3	ID1	51	13	2.308	2.91E-06
#3	H3F3B	100	31	2.305	9.72E-30
#3	RPL6	98	35	2.303	4.29E-27
#3	RPL11	98	37	2.297	2.65E-27
#3	RPL7A	95	33	2.295	7.75E-26
#3	CLDN4	92	26	2.293	3.64E-24
#3	QDPR	98	25	2.29	3.82E-29
#3	LMX1A	93	17	2.287	9.1E-28
#3	PKM	98	45	2.276	1.76E-28
#3	HIST1H2BK	88	22	2.272	9.54E-21
#3	MUT	88	18	2.269	7.68E-25
#3	RPL3	100	37	2.265	1.9E-27
#3	RPL9	100	39	2.264	5.35E-26
#3	MTRNR2L1	97	47	2.262	1.26E-21
#3	CISD1	98	27	2.259	3.1E-32
#3	CD63	95	34	2.257	4.11E-25
#3	RPS27A	93	35	2.254	1.37E-26
#3	RPS15	98	33	2.248	2.66E-29
#3	FXYD3	97	33	2.248	1.38E-26
#3	TUBA1A	97	26	2.234	2.44E-26
#3	RPL4	98	36	2.229	9.23E-27
#3	HLA-E	81	27	2.228	1.18E-17
#3	CAST	95	30	2.218	1.5E-21
#3	TMBIM6	97	32	2.215	2.03E-27
#3	ADH5	90	25	2.215	1.05E-22
#3	RPL27A	98	37	2.214	2.52E-28
#3	VMP1	88	26	2.21	4.47E-20

#3	RPS13	95	33	2.207	6.09E-23
#3	CAMK2N1	97	34	2.206	1.97E-27
#3	RPL10	98	36	2.204	1.1E-26
#3	HINT1	93	26	2.201	2.73E-24
#3	RPS16	97	33	2.2	2.22E-27
#3	DSTN	93	25	2.197	7.08E-22
#3	RPS9	98	30	2.195	9.79E-27
#3	EIF1	95	31	2.195	1.24E-25
#3	RPS28	98	35	2.194	9.14E-27
#3	S100A16	93	24	2.189	2.8E-20
#3	RAB3C	81	15	2.184	1.41E-18
#3	H19	32	7	2.184	0.00013
#3	RPS24	100	40	2.18	1.6E-26
#3	SH3BGRL3	98	39	2.179	2.73E-24
#3	RPL10A	97	33	2.178	5.1E-28
#3	DBI	90	26	2.178	1.14E-24
#3	PCSK1	88	26	2.178	8.32E-17
#3	MTRNR2L8	78	37	2.176	1.11E-14
#3	YBX1	97	33	2.174	8.55E-24
#3	RFX6	90	23	2.174	2.02E-23
#3	XBP1	81	19	2.174	1.98E-21
#3	ADGRG4	63	8	2.171	2.15E-15
#3	RPLP2	98	34	2.169	1.56E-28
#3	GNB2L1	98	32	2.168	2E-27
#3	RPL5	98	38	2.164	1.39E-28
#3	RPL18	97	31	2.162	1.95E-26
#3	CD24	98	41	2.161	4.35E-25
#3	RPS6	100	44	2.157	1.08E-25
#3	APP	95	29	2.157	5.03E-25
#3	RPL28	98	35	2.155	1.56E-27
#3	UBC	97	30	2.155	8E-27
#3	FOSB	83	21	2.155	5.31E-19
#3	MYL6	98	35	2.148	4.14E-26
#3	COX8A	93	28	2.147	4.26E-24
#3	SSR4	97	28	2.144	1.76E-25
#3	ATP6V1B2	95	22	2.142	3.8E-26
#3	EPCAM	98	38	2.141	6.08E-25
#3	MAPKAPK2	95	25	2.138	1.06E-25
#3	GABARAP	100	37	2.13	1.06E-30
#3	RPS15A	98	32	2.128	1.09E-27
#3	TUBA1B	90	24	2.128	1.36E-26
#3	GNG2	95	20	2.124	5.23E-29
#3	PLAC8	90	25	2.124	4.94E-24
#3	GPR160	93	24	2.123	1.97E-23
#3	RPL18A	100	44	2.12	1.45E-26
#3	HSP90AA1	97	35	2.12	7.53E-26
#3	PKDCC	88	19	2.12	1.34E-20
#3	RPL30	98	35	2.119	2.43E-29
#3	RPL19	97	30	2.119	3.64E-26
#3	RPS23	98	32	2.118	3.71E-27
#3	SERF2	97	35	2.118	3.18E-25
#3	COX4I1	97	33	2.117	2.14E-26
#3	HSPA5	90	27	2.117	1.04E-19
#3	RPL8	98	30	2.113	1.19E-26
#3	FTH1	98	34	2.112	5.11E-27
#3	LRP10	92	24	2.108	6.62E-23
#3	PROM1	98	28	2.107	7.03E-27
#3	CD59	97	28	2.106	3.82E-24
#3	RPL26	98	35	2.105	5.77E-28
#3	ABHD2	93	26	2.103	1.45E-23
#3	RPL37	97	39	2.1	9.23E-26
#3	HSP90AB1	98	34	2.099	2.62E-26
#3	RPS25	100	31	2.094	2.53E-26
#3	MDK	100	44	2.092	1.31E-26
#3	GOLM1	95	25	2.092	1.09E-16
#3	RPS19	98	32	2.087	7.77E-25
#3	RPS4X	97	32	2.082	9.85E-26
#3	CALM1	98	36	2.08	6.9E-26
#3	H1FX	93	26	2.078	2.87E-23
#3	PGK1	90	23	2.077	4.93E-22
#3	KRT19	97	35	2.076	1.63E-24
#3	IDI1	85	18	2.076	3.7E-24
#3	RPLP0	95	29	2.075	1.24E-25
#3	RPL7	98	32	2.071	9.48E-25
#3	TMSB10	98	39	2.07	3.1E-28
#3	RPS18	93	31	2.07	1.07E-24
#3	S100A11	100	40	2.065	1.17E-28
#3	COX7A2	93	27	2.062	1.5E-22
#3	CFL1	100	53	2.06	1.86E-24
#3	CITED4	85	19	2.058	1.54E-18
#3	ATP5A1	97	26	2.056	1.89E-24
#3	RPS20	98	41	2.055	1.34E-25
#3	CALM2	98	34	2.055	1.16E-23
#3	FAU	97	30	2.055	7.04E-26
#3	TMEM59	92	27	2.054	7.4E-24
#3	RPS7	97	32	2.052	5.19E-24
#3	FOLH1	63	11	2.051	1.57E-12
#3	CLTC	95	25	2.049	2.96E-28
#3	JTB	92	22	2.047	9.36E-26
#3	ECH1	97	23	2.045	4.2E-26
#3	RPL34	98	37	2.042	4.68E-25
#3	EEF2	97	35	2.038	2.43E-27
#3	RPL14	98	35	2.034	1.8E-25
#3	ACTB	98	41	2.033	2.13E-24
#3	KRT18	92	27	2.033	2.59E-20
#3	RPL32	98	31	2.032	4.19E-29
#3	ZFP36	81	16	2.032	1.88E-19
#3	LDHA	66	20	2.032	2.57E-13
#3	ATP5J2	97	28	2.028	1.58E-28
#3	EEF1B2	95	27	2.028	1.49E-25
#3	ATP5L	93	25	2.024	1.22E-24
#3	BTG2	68	17	2.024	4.34E-14
#3	PPIA	97	32	2.02	1.33E-23
#3	CD164	95	27	2.02	4.11E-26
#3	TFPI2	44	5	2.016	2.74E-10
#3	DUSP1	81	18	2.015	9.24E-22
#3	VWA5B2	90	25	2.012	3.04E-21
#3	RPL12	97	34	2.011	1.29E-22
#3	YWHAB	97	34	2.01	1.13E-26
#3	OAZ1	97	27	2.007	2.38E-28
#3	RPL36	95	30	2.007	8.88E-25
#3	RAB26	92	25	2.002	6.67E-24
#3	RAB27A	90	17	2.001	1.22E-25

#3	RPL23	97	33	2	5.31E-24
#3	PFN1	98	39	1.999	6.7E-25
#3	BEX1	85	16	1.998	1.6E-21
#3	PSAP	93	27	1.997	1.32E-23
#3	PRKAR1A	93	28	1.99	3.2E-23
#3	SQSTM1	92	25	1.986	2.55E-20
#3	GUCY2C	85	18	1.984	1.79E-19
#3	COX6C	97	27	1.978	1.91E-25
#3	TTC3	93	27	1.97	5E-20
#3	DDX5	95	32	1.969	6.97E-28
#3	GPX4	97	29	1.966	3.35E-29
#3	DYNLL1	93	26	1.966	4.51E-24
#3	RPL35A	95	30	1.965	1.39E-24
#3	CAPNS1	93	35	1.965	1.83E-25
#3	UCP2	95	28	1.964	1.09E-25
#3	RTN4	97	30	1.963	1.15E-26
#3	DSP	98	33	1.961	3.24E-26
#3	RPL35	98	32	1.96	2.73E-25
#3	ARPC2	97	33	1.957	9.86E-24
#3	RPS5	97	34	1.954	4.32E-25
#3	CST3	95	33	1.954	1.3E-22
#3	ID3	27	5	1.952	0.00006
#3	FKBP1A	93	30	1.947	1.35E-25
#3	SCG3	92	23	1.947	9.34E-21
#3	C12ORF75	93	21	1.946	5.86E-22
#3	RPL22	95	29	1.943	5.66E-25
#3	NDUFC2	86	20	1.943	1.38E-23
#3	CDH17	86	25	1.942	1.88E-18
#3	UBA52	97	31	1.941	9.97E-27
#3	RPL23A	92	30	1.94	2.32E-22
#3	EIF4A2	97	26	1.934	2.7E-24
#3	C1QL1	75	22	1.93	6.09E-11
#3	INSIG1	69	18	1.927	3.57E-13
#3	MNX1	78	16	1.925	4.02E-18
#3	RPL17	95	28	1.924	2.11E-25
#3	CACNA1A	98	33	1.923	4.16E-25
#3	EID1	95	26	1.922	2.9E-22
#3	RPL38	95	33	1.921	5.77E-22
#3	TSPAN3	88	27	1.921	1.3E-17
#3	RPL24	97	29	1.918	2.07E-23
#3	MICAL2	85	20	1.916	1.87E-17
#3	RAB11A	98	26	1.915	1.98E-20
#3	CLIC1	92	26	1.912	4.31E-26
#3	SRP9	95	27	1.908	1.95E-26
#3	MTRNR2L2	68	16	1.907	6.74E-16
#3	TPI1	86	25	1.906	2.27E-18
#3	IDS	95	27	1.904	4.09E-24
#3	CSTB	90	26	1.904	4.51E-20
#3	MRFAP1	90	25	1.904	1.05E-19
#3	TM9SF3	93	27	1.903	5.52E-24
#3	TIMP1	81	24	1.903	4.13E-18
#3	PCSK2	68	13	1.902	2.11E-16
#3	PTMS	95	28	1.901	3.11E-30
#3	ATP5B	95	29	1.9	3.54E-25
#3	SOAT1	93	22	1.896	4.17E-25
#3	PTMA	100	36	1.891	4.62E-24
#3	ARF1	95	25	1.888	1.15E-25
#3	SLC7A2	86	13	1.888	6.72E-20
#3	EIF4G2	93	33	1.885	1.5E-21
#3	SLC25A3	90	26	1.884	6.91E-18
#3	RNASE4	90	17	1.883	1.08E-25
#3	ACTN4	100	38	1.881	4.55E-28
#3	TM4SF5	92	21	1.878	1.21E-20
#3	EMP1	56	6	1.878	3.4E-11
#3	ATP5G2	95	25	1.875	1.4E-23
#3	EMC10	90	24	1.875	7.56E-17
#3	HNRNPC	93	27	1.874	1.94E-25
#3	RPL31	95	28	1.872	1.1E-22
#3	TXN	90	29	1.872	6.41E-19
#3	RHOA	93	28	1.871	2.84E-23
#3	MCL1	93	20	1.87	1.65E-23
#3	HLA-C	95	29	1.869	4.89E-23
#3	YWHAE	93	27	1.864	1.01E-24
#3	ARPC3	88	24	1.862	5.97E-22
#3	RPL27	97	29	1.859	7.95E-23
#3	GNB1	93	26	1.856	6.81E-25
#3	SERPINB1	92	23	1.856	2.11E-19
#3	CYSTM1	90	27	1.856	1.57E-22
#3	RDH11	95	24	1.852	1.14E-20
#3	HIGD1A	90	22	1.851	7.76E-21
#3	TNFRSF11B	39	5	1.851	5.9E-08
#3	AES	93	29	1.849	1.73E-25
#3	ROMO1	93	23	1.849	1.15E-20
#3	HSP90B1	95	28	1.847	1.48E-20
#3	PCBP1	88	24	1.844	2.64E-18
#3	CALY	85	22	1.844	3.21E-16
#3	COX7C	97	30	1.843	1.02E-24
#3	CLDN3	88	21	1.842	1.02E-18
#3	MALAT1	98	55	1.841	9.87E-20
#3	SLC38A11	81	15	1.839	1.87E-17
#3	RAC1	98	32	1.837	8.59E-27
#3	ATP6V0B	93	24	1.834	3.89E-23
#3	SURF4	88	21	1.834	2.1E-21
#3	C2ORF54	75	16	1.834	3.56E-17
#3	ANXA10	68	19	1.83	1.11E-12
#3	BNIP3	80	19	1.829	3.11E-17
#3	CBX6	93	25	1.828	1E-23
#3	ACADVL	93	25	1.828	1.99E-21
#3	FLVCR1	88	21	1.828	1.66E-21
#3	TM9SF2	92	23	1.827	6.31E-22
#3	CHCHD2	93	27	1.826	2.98E-22
#3	TSPAN13	88	23	1.826	1.69E-20
#3	ATP2B4	88	15	1.825	8.31E-19
#3	HNRNPA2B1	95	30	1.823	1.19E-21
#3	SARAF	66	11	1.822	1.08E-17
#3	RPL39	97	29	1.82	4.17E-27
#3	ELF3	85	23	1.818	1.8E-17
#3	HEPACAM2	92	24	1.817	3.98E-18
#3	ATP5G3	90	23	1.817	1.17E-18
#3	CAMK2B	97	21	1.816	3.58E-24
#3	SPINT2	100	46	1.815	1.18E-23
#3	DNAJC15	86	19	1.813	1.4E-16
#3	MPC2	92	18	1.811	7.7E-23

#3	ATP6V0C	93	29	1.802	2.55E-22
#3	FOXA1	85	19	1.799	6.69E-19
#3	CD9	78	19	1.799	6.02E-19
#3	SERBP1	92	24	1.798	2.95E-22
#3	NKX2-2	90	28	1.796	3.75E-20
#3	RHEB	93	19	1.795	6.37E-18
#3	HDLBP	95	26	1.793	8.18E-22
#3	ENDOD1	71	14	1.793	2.51E-18
#3	UBL5	93	25	1.792	1.73E-26
#3	PRDX5	97	32	1.789	6.02E-25
#3	MYO10	81	20	1.788	8.75E-19
#3	HLA-A	92	32	1.787	1.32E-22
#3	STARD10	95	26	1.785	5.95E-23
#3	TOMM7	88	19	1.782	1.26E-18
#3	RNF128	81	23	1.782	3.57E-15
#3	ST6GALNAC4	81	15	1.781	4.8E-20
#3	ERBB3	95	25	1.778	6.02E-22
#3	MYL12B	93	27	1.778	3.76E-21
#3	NDUFA1	92	26	1.777	3.91E-20
#3	QPCT	80	18	1.776	4.46E-18
#3	LRRC75A-AS1	61	11	1.776	2.21E-15
#3	ITM2B	93	31	1.775	1.04E-20
#3	FOXP1	90	26	1.774	3.06E-20
#3	C1ORF43	98	23	1.772	2.61E-17
#3	YWHAQ	95	26	1.771	5.52E-24
#3	MSMO1	92	26	1.769	2.91E-19
#3	HTATSF1	80	17	1.764	3.89E-16
#3	GLUL	78	20	1.764	6.85E-19
#3	COX7A2L	85	22	1.758	2.6E-22
#3	FDFT1	85	22	1.758	1.9E-17
#3	SYT7	85	23	1.758	2.08E-17
#3	BRK1	90	23	1.756	1.05E-19
#3	NGFRAP1	93	24	1.754	5.31E-19
#3	ATP1B3	85	20	1.751	2.37E-24
#3	PAPSS2	78	13	1.751	5.31E-15
#3	SSR1	81	22	1.747	6.69E-17
#3	BSG	95	25	1.745	6.57E-21
#3	ATP9A	88	19	1.745	9.39E-20
#3	SEC62	100	28	1.744	1.52E-26
#3	S100A13	88	20	1.744	2.83E-25
#3	UQCR11	95	24	1.743	1.51E-21
#3	YWHAZ	95	35	1.742	1.1E-21
#3	CMTM6	90	24	1.74	4.63E-17
#3	SEC11C	86	24	1.74	7.2E-18
#3	NDUFB8	86	18	1.739	3.04E-21
#3	P4HB	86	25	1.736	4.06E-22
#3	CANX	86	28	1.734	8.64E-21
#3	ALDOA	85	25	1.734	1.02E-12
#3	JUND	93	28	1.733	4.4E-21
#3	ATP2A2	86	21	1.733	1.08E-24
#3	GCLM	88	22	1.73	1.46E-20
#3	HNRNPK	88	27	1.73	1.14E-18
#3	CD55	58	13	1.73	1.2E-06
#3	NDFIP1	80	18	1.729	1.74E-18
#3	MARCKSL1	93	29	1.727	6.96E-24
#3	OST4	81	20	1.724	2.34E-22
#3	SERPINA1	83	25	1.723	2.49E-13
#3	MGLL	86	23	1.721	1.53E-14
#3	ATP6V1G1	92	24	1.72	3.7E-19
#3	UQCRQ	95	28	1.718	4E-23
#3	PDZRN3	76	14	1.716	3.79E-16
#3	KLK11	73	14	1.716	2.86E-14
#3	ATPIF1	92	24	1.715	1.57E-18
#3	USMG5	90	22	1.715	1.18E-18
#3	PLP2	86	19	1.713	2.06E-16
#3	HDGF	92	27	1.711	3.71E-20
#3	STX1A	90	20	1.711	1.5E-24
#3	37500	86	22	1.711	7.29E-20
#3	ARPP19	90	22	1.71	3.48E-18
#3	ARPC5	90	23	1.708	4.08E-20
#3	HIST1H1C	85	19	1.708	6.32E-17
#3	TMEM176B	83	25	1.708	6.09E-15
#3	CSDE1	97	29	1.707	3.58E-27
#3	RBM3	95	25	1.707	3.48E-23
#3	38047	76	12	1.707	4.58E-19
#3	SELK	71	15	1.706	4.8E-20
#3	SEPW1	93	29	1.705	5.05E-20
#3	MIR7-3HG	81	15	1.699	3.37E-15
#3	LMNA	85	24	1.698	5.31E-22
#3	CYCS	83	23	1.698	1.81E-16
#3	CDC42EP3	80	17	1.698	4.95E-17
#3	RASD1	54	14	1.698	1.63E-07
#3	PTBP3	90	23	1.695	9.88E-21
#3	USO1	78	18	1.695	2.13E-15
#3	UBA1	95	27	1.692	2.26E-25
#3	ARF4	86	20	1.692	1.15E-16
#3	SEC61B	90	22	1.691	1.57E-24
#3	MARCKS	90	30	1.691	1.91E-22
#3	RAB32	68	8	1.691	2.22E-13
#3	COX7B	93	24	1.69	1.23E-22
#3	HSD17B12	86	21	1.69	6.89E-22
#3	PAX4	71	18	1.69	1.22E-13
#3	RPL36AL	90	24	1.688	6.54E-19
#3	SC5D	56	8	1.686	1.42E-13
#3	MIA3	78	18	1.684	9.18E-15
#3	PCBD1	83	21	1.682	6.42E-21
#3	ATP5J	92	26	1.681	1.77E-17
#3	RASSF6	86	22	1.681	3.09E-19
#3	MORF4L1	93	27	1.68	2.83E-20
#3	KIAA1324	83	21	1.676	7E-21
#3	SMC4	80	15	1.676	2.28E-19
#3	ITGB1	88	27	1.674	8.8E-18
#3	PTGES3	92	23	1.673	1.01E-18
#3	NDUFB11	93	24	1.669	2.58E-25
#3	SEC61G	83	23	1.666	9.47E-20
#3	BZW1	83	21	1.666	3.21E-19
#3	PARK7	93	21	1.665	6.48E-19
#3	COX17	83	19	1.665	1.21E-15
#3	SYNGR2	88	20	1.664	3.24E-22
#3	TM4SF4	86	29	1.661	4.45E-13
#3	SCG2	78	28	1.661	4.18E-11
#3	PPDPF	97	31	1.658	8.23E-22
#3	NR4A2	47	14	1.657	0.00004

#3	SMIM6	86	18	1.656	2.27E-16
#3	MYH9	90	29	1.654	8.31E-21
#3	CAMK2D	78	16	1.654	5.45E-21
#3	ABCC3	83	20	1.653	4.5E-20
#3	TMA7	88	25	1.652	2.96E-22
#3	NDUFA13	92	23	1.651	8.52E-25
#3	RAB2A	88	22	1.649	1.33E-15
#3	ZKSCAN1	93	25	1.648	5.67E-21
#3	PFN2	92	23	1.648	2.96E-21
#3	H1F0	80	19	1.646	2.78E-16
#3	PRDX2	93	22	1.644	2.14E-21
#3	EMP2	64	16	1.644	2.15E-09
#3	FAM134B	85	19	1.642	1.52E-17
#3	SLC3A2	80	15	1.642	3.35E-15
#3	TOB1	71	15	1.641	2.01E-13
#3	EVPL	76	16	1.638	1.46E-21
#3	PPHLN1	68	14	1.636	1.2E-13
#3	HMGN1	88	26	1.635	1.76E-21
#3	PRDX3	75	17	1.634	1.24E-13
#3	UQCRFS1	73	16	1.634	1.51E-14
#3	WSB2	93	21	1.63	1.5E-24
#3	ARL14	71	17	1.63	4.61E-14
#3	ZAK	85	18	1.629	1.38E-23
#3	RANBP2	85	20	1.625	3.37E-16
#3	TP53INP2	83	19	1.624	9.15E-19
#3	PSMB4	85	20	1.622	8.2E-16
#3	COX5B	80	23	1.622	1.18E-16
#3	IL32	49	15	1.622	0.00002
#3	SET	95	27	1.621	5.97E-22
#3	JUN	59	15	1.621	1.1E-08
#3	HIPK2	88	25	1.62	4.25E-18
#3	GNAI2	95	28	1.619	2.13E-20
#3	PDXDC1	83	20	1.619	1.21E-20
#3	COX6B1	86	24	1.618	6.9E-19
#3	FAM120A	88	24	1.616	1.22E-15
#3	NDUFB1	88	18	1.614	5.35E-21
#3	PTP4A2	88	25	1.613	5.31E-19
#3	SLC44A1	78	20	1.613	2.82E-15
#3	HEPH	64	10	1.613	2.88E-14
#3	RAB1A	86	20	1.612	1.04E-19
#3	ID2	56	11	1.612	1.55E-10
#3	ATP6AP1	86	20	1.611	1.54E-18
#3	ACTR2	92	23	1.609	1.06E-21
#3	ATP1B1	78	25	1.609	5.15E-15
#3	SCP2	75	18	1.609	1.64E-12
#3	TMEM139	78	14	1.605	1.3E-15
#3	CDC42	90	25	1.603	8.26E-23
#3	WFS1	85	18	1.603	1.02E-18
#3	SPTBN1	83	24	1.602	1.68E-17
#3	GHITM	81	18	1.601	5.39E-12
#3	RPL29	92	24	1.599	2.43E-18
#3	DYNLRB1	95	29	1.597	2.65E-22
#3	ATP2A3	81	19	1.596	1.13E-14
#3	CEACAM5	36	12	1.596	0.00041
#3	MTRNR2L9	54	7	1.594	2.59E-11
#3	VCP	86	22	1.593	1.07E-18
#3	RPS27L	86	26	1.591	2.29E-18
#3	MLF2	88	25	1.59	3.6E-21
#3	CAPZA2	86	21	1.59	1.06E-21
#3	RPL13A	95	25	1.588	5.57E-20
#3	TAPBP	88	21	1.588	1.83E-20
#3	CYB5R3	92	24	1.587	8.64E-22
#3	TMED10	86	19	1.587	5.71E-17
#3	MUC2	27	7	1.587	0.0002
#3	SKP1	90	23	1.586	1.91E-19
#3	CYP51A1	97	25	1.584	2.59E-23
#3	CORO1B	80	19	1.582	4.76E-16
#3	TBCA	81	19	1.581	1.38E-16
#3	BTF3	92	21	1.58	1.03E-18
#3	ACLY	90	23	1.58	8.75E-17
#3	MAGED1	88	20	1.58	3.92E-19
#3	UBXN4	86	27	1.58	3.43E-18
#3	ASCL2	71	11	1.58	5.33E-14
#3	EGR3	49	8	1.579	2.61E-11
#3	LYZ	83	32	1.577	1.07E-12
#3	SGPL1	81	18	1.577	1.68E-17
#3	MYADM	85	24	1.576	2.64E-16
#3	ETS2	76	19	1.576	2.41E-14
#3	PPP1CB	86	24	1.574	1.33E-13
#3	SEC61A1	85	20	1.574	3.79E-18
#3	HNRNPU	86	26	1.572	2.06E-21
#3	LGALS3BP	86	24	1.572	1.51E-17
#3	IER3	53	12	1.572	1.54E-08
#3	SGK223	73	12	1.571	2.92E-12
#3	HGD	64	12	1.571	9.37E-13
#3	EDF1	95	25	1.57	6.27E-18
#3	GPI	85	26	1.568	9.63E-13
#3	GCLC	81	13	1.568	1.49E-19
#3	PFDN5	92	23	1.567	4.7E-20
#3	SORD	85	17	1.566	1.11E-17
#3	PRR15L	78	11	1.566	3.64E-19
#3	FADS2	86	19	1.565	2.78E-16
#3	CD74	83	26	1.565	6.72E-15
#3	GPD1	73	11	1.564	4.32E-16
#3	DSC2	92	20	1.563	6.19E-22
#3	HSPE1	85	20	1.562	2.67E-20
#3	LLGL2	85	23	1.56	6.59E-17
#3	SRP14	88	27	1.559	7.5E-19
#3	COPE	85	21	1.559	1.78E-16
#3	ARL6IP5	81	18	1.558	1.94E-17
#3	NDUFS5	92	22	1.556	9.89E-22
#3	PPAPDC1B	76	17	1.556	3.89E-16
#3	KDELR2	76	21	1.555	3.9E-14
#3	PSMB1	80	21	1.554	3.78E-20
#3	PLEKHB1	90	20	1.553	1.07E-14
#3	PSME4	80	15	1.553	2.23E-13
#3	BAIAP3	81	17	1.551	5.17E-17
#3	NDUFB2	86	20	1.55	4.14E-15
#3	LPP	90	23	1.549	4.01E-18
#3	JUP	85	23	1.549	1.66E-14
#3	PSMA7	93	26	1.548	8.96E-23
#3	GABARAPL2	88	19	1.548	1.82E-22
#3	BNIP3L	81	19	1.548	2.81E-17

#3	CADM2	66	8	1.547	5.33E-12
#3	FASN	86	20	1.545	2.26E-20
#3	ZNF664	80	22	1.545	2.07E-16
#3	BAALC	75	12	1.545	2.09E-14
#3	SELENBP1	90	21	1.543	1.08E-25
#3	SUMO2	92	26	1.542	4.37E-22
#3	PPL	76	15	1.541	8.09E-15
#3	SRPR	76	15	1.541	2.67E-12
#3	PSMD8	83	19	1.539	1.94E-20
#3	SCN3A	80	14	1.539	3.09E-14
#3	KIAA1598	73	18	1.539	2.57E-13
#3	H2AFY	88	26	1.535	2.01E-19
#3	POLR2L	92	25	1.534	9.58E-18
#3	ATF3	66	12	1.533	4.01E-10
#3	MDH2	83	16	1.532	2.95E-20
#3	RAB1B	83	22	1.531	6.48E-19
#3	GC	81	22	1.531	1.05E-12
#3	SAP18	75	20	1.531	1.1E-15
#3	LOC389332	76	15	1.53	1.94E-17
#3	ANXA6	80	16	1.528	6.2E-19
#3	AKAP9	85	25	1.526	3.66E-14
#3	CTDSPL	81	19	1.526	6.67E-15
#3	BCAR3	76	18	1.526	8.31E-13
#3	DYNLT1	83	20	1.525	1.72E-17
#3	LFNG	81	22	1.521	2.37E-16
#3	MAPK14	83	14	1.52	1.82E-20
#3	ZYX	76	21	1.52	2.71E-12
#3	GDI2	85	20	1.517	6.07E-15
#3	EIF3L	80	20	1.517	1.7E-16
#3	EI24	85	24	1.516	5.54E-16
#3	ST13	83	19	1.516	1.68E-17
#3	ADH6	66	12	1.516	9.79E-14
#3	NAP1L1	93	24	1.515	2.08E-21
#3	SPCS1	75	17	1.514	7.48E-14
#3	ALDOC	83	16	1.513	3.15E-19
#3	SMPD3	76	15	1.513	3.23E-17
#3	SLC7A8	73	12	1.513	1.97E-14
#3	KPNA6	93	22	1.512	1.58E-18
#3	SREBF2	69	12	1.511	7.62E-13
#3	CAP1	88	26	1.51	3.32E-15
#3	SRP19	76	20	1.51	4.37E-13
#3	FDPS	71	14	1.51	1.34E-13
#3	RGS16	49	8	1.51	1.1E-07
#3	PABPC1	86	28	1.508	1.91E-18
#3	SSR2	85	21	1.507	2.25E-15
#3	ERP29	80	19	1.507	1.36E-14
#3	GNAQ	61	10	1.506	1.82E-13
#3	GDF15	44	16	1.505	6.82E-08
#3	TGOLN2	86	24	1.504	1.73E-19
#3	TECR	83	17	1.504	7.46E-17
#3	JAK1	80	19	1.503	2.82E-13
#3	WAC	88	21	1.502	8.19E-17
#3	TPD52	86	24	1.502	1.15E-13
#3	AP2M1	86	20	1.501	4.09E-16
#3	DPP4	75	14	1.5	1.51E-14
#3	CCNI	88	25	1.499	7.8E-21
#3	PCBP2	88	24	1.499	1.16E-19
#3	KIF5B	86	24	1.499	4.08E-13
#3	PTPRN2	83	19	1.499	1.14E-18
#3	EIF4B	83	24	1.498	1.18E-18
#3	HMGCR	83	18	1.497	4.47E-17
#3	NFATC2IP	88	22	1.496	5.63E-17
#3	SSBP3	81	19	1.496	1.24E-14
#3	ATP5G1	80	19	1.496	1.27E-16
#3	FAM46B	58	9	1.496	3.44E-12
#3	GDI1	90	22	1.495	3.38E-17
#3	ZNF706	83	20	1.495	1.76E-13
#3	SMDT1	63	9	1.495	7.55E-16
#3	ABCA5	90	24	1.494	8.51E-21
#3	SERPINB6	86	25	1.494	8.57E-19
#3	PHF20L1	75	20	1.494	8.07E-14
#3	SLC25A39	80	21	1.491	4.14E-12
#3	CADPS	90	25	1.49	2.92E-19
#3	PPA1	86	15	1.49	1.55E-14
#3	EIF2S2	80	22	1.49	2.81E-18
#3	NDUFB9	75	16	1.49	6.88E-14
#3	CLDN18	44	7	1.489	4.67E-07
#3	CALR	88	26	1.488	1.48E-18
#3	EIF4H	83	24	1.488	3.79E-18
#3	MYO1D	73	18	1.487	1.34E-13
#3	DYNC1H1	90	22	1.485	3.76E-19
#3	STIM2	68	15	1.485	8.38E-14
#3	CALM3	88	20	1.484	2.79E-22
#3	MGST3	86	18	1.484	6.6E-18
#3	CCNY	80	17	1.484	2.13E-15
#3	RIMBP2	93	23	1.482	2.29E-22
#3	XRCC5	88	25	1.482	5.65E-17
#3	S100A10	85	29	1.481	8.83E-13
#3	CDH1	88	24	1.48	2.65E-16
#3	SCARB2	83	23	1.48	1.11E-17
#3	ZFAND5	75	18	1.479	7.52E-15
#3	GPX1	86	20	1.478	1.7E-16
#3	ATP6V1A	80	21	1.478	1.78E-16
#3	G3BP2	90	19	1.477	4.94E-18
#3	IRF2BP2	85	22	1.477	4.59E-16
#3	CCT8	76	18	1.477	7.48E-14
#3	FAM46A	76	22	1.477	7.31E-12
#3	UBE2L3	81	19	1.476	2.32E-15
#3	ARF3	85	21	1.474	9.03E-16
#3	NPTN	80	18	1.474	6.9E-13
#3	PERP	83	23	1.473	2.58E-20
#3	DNAJC12	66	15	1.473	2.01E-10
#3	ANXA2	81	25	1.472	1.48E-12
#3	RPN2	78	20	1.472	9.95E-16
#3	CCND1	51	14	1.472	3.48E-08
#3	SELT	88	20	1.471	4.12E-17
#3	CD46	83	19	1.471	2.07E-16
#3	SLC38A10	78	17	1.471	1.02E-12
#3	RGS4	68	18	1.471	7.18E-11
#3	APLP2	85	24	1.47	1.87E-14
#3	PFKP	73	17	1.47	1.43E-15
#3	PPP6R1	81	18	1.469	6.09E-17
#3	RPS4Y1	71	9	1.469	1.41E-19

#3	EPHX1	68	15	1.469	5.77E-10
#3	SERP1	86	21	1.468	8.07E-14
#3	FAM49B	73	15	1.465	8.09E-15
#3	ODC1	71	11	1.465	4.51E-13
#3	ACSL4	64	14	1.465	3.24E-13
#3	PTGES2	75	16	1.464	4.49E-17
#3	EFHD2	88	23	1.463	3.41E-17
#3	MATR3	86	22	1.462	4.8E-19
#3	TMEM30A	83	21	1.462	3.86E-14
#3	CDKN2A	76	22	1.462	8.73E-15
#3	CTNNA1	75	19	1.462	1.33E-13
#3	ELL2	81	22	1.461	1.6E-13
#3	DDX17	90	27	1.46	6.41E-21
#3	RAB7A	80	20	1.46	2.29E-16
#3	ARFGEF3	61	9	1.46	1.28E-12
#3	CD151	83	23	1.458	4.7E-15
#3	LPCAT3	76	19	1.458	8.31E-15
#3	LAD1	75	20	1.457	5.83E-10
#3	EIF5B	90	26	1.456	6.74E-15
#3	ALKBH5	76	19	1.456	9.98E-14
#3	LIPG	54	9	1.456	1.09E-09
#3	MKNK2	78	19	1.455	1.37E-14
#3	LXN	58	6	1.455	1.83E-08
#3	NDUFA11	88	20	1.454	5.52E-15
#3	TM7SF2	81	19	1.454	2.81E-17
#3	GSN	73	21	1.454	2.32E-10
#3	MYO6	90	27	1.452	1.17E-15
#3	ECHS1	85	18	1.452	3.5E-18
#3	WASL	80	16	1.452	4.29E-20
#3	ALDH2	71	15	1.452	3.96E-16
#3	FFAR2	59	14	1.451	1.66E-10
#3	ARPC1B	75	21	1.45	2.14E-15
#3	SLC25A1	83	22	1.449	3.74E-16
#3	XRCC6	80	17	1.449	7.48E-14
#3	SLC7A5	64	11	1.448	2.14E-13
#3	NEURL1	59	9	1.448	1.56E-13
#3	PHLDA2	49	11	1.448	1.97E-09
#3	CLTA	85	17	1.447	5.58E-15
#3	NONO	92	23	1.446	1.28E-17
#3	KLF6	83	24	1.446	1.02E-13
#3	MS4A8B	29	16	1.445	0.00204
#3	PHPT1	90	19	1.444	1.13E-14
#3	LGMN	68	18	1.444	5.21E-13
#3	UQCRC1	81	17	1.443	7.86E-21
#3	EIF3H	78	18	1.443	7.48E-14
#3	NDUFAB1	76	19	1.443	4.71E-15
#3	ATP2B1	78	19	1.441	3.2E-14
#3	PDLIM5	76	22	1.441	4.55E-12
#3	HK2	63	8	1.441	4.23E-15
#3	SON	88	27	1.44	7.71E-15
#3	MFSD6	73	14	1.438	4.69E-16
#3	GMDS	73	18	1.437	1.49E-12
#3	OCIAD1	86	17	1.436	5.04E-11
#3	TMEM176A	81	20	1.436	2.87E-16
#3	RAP1GAP2	78	22	1.436	1.85E-15
#3	MAPK1	73	14	1.435	8.13E-13
#3	YIPF3	85	19	1.434	9.58E-16
#3	ALDH1A1	76	21	1.434	1.11E-12
#3	TAF9	61	13	1.434	6.4E-09
#3	EIF5	85	24	1.433	1.48E-13
#3	PBXIP1	85	21	1.432	1.49E-13
#3	TM4SF1	63	18	1.432	1.76E-07
#3	KPNB1	88	22	1.431	1.07E-18
#3	CUTA	85	22	1.429	6.84E-18
#3	HMGN3	85	19	1.429	2.25E-12
#3	PLXNB1	81	22	1.429	4.68E-12
#3	C11ORF58	76	22	1.425	2.51E-17
#3	PEG10	49	13	1.424	4.12E-07
#3	MAPK3	85	19	1.423	1.88E-16
#3	LUC7L3	88	27	1.421	1.69E-14
#3	TXNIP	83	18	1.421	1.1E-11
#3	RHOB	73	12	1.421	2.22E-16
#3	CD81	85	25	1.42	3.52E-14
#3	SF3B5	85	19	1.419	4.56E-15
#3	RUNDC3A	75	18	1.419	9.3E-15
#3	PLA2G12A	85	17	1.418	2.48E-15
#3	HSBP1	81	22	1.418	8.04E-16
#3	PSMD1	73	18	1.418	1.55E-14
#3	CMPK1	83	22	1.417	6.07E-17
#3	RAD23B	78	19	1.417	1.01E-14
#3	IL13RA1	78	20	1.416	2.71E-19
#3	ANAPC16	75	17	1.415	3.44E-14
#3	CAMK2G	63	10	1.415	1.52E-10
#3	HES1	39	7	1.414	2.76E-09
#3	MFGE8	75	16	1.412	2.32E-14
#3	FLOT1	80	21	1.41	1.77E-15
#3	PDLIM1	75	22	1.41	5.05E-12
#3	TCEB2	85	25	1.409	3.9E-18
#3	PSMB5	75	16	1.409	4.58E-15
#3	PRKCH	69	11	1.409	2.55E-16
#3	CAPN8	66	13	1.409	1.32E-11
#3	VDAC1	90	28	1.408	2.39E-16
#3	DECR1	81	17	1.408	6.36E-13
#3	AK1	90	21	1.406	6.71E-14
#3	RTN3	81	22	1.406	1.57E-19
#3	LINC00657	86	21	1.405	1.55E-15
#3	C14ORF2	81	21	1.405	2.71E-19
#3	IDH1	85	19	1.401	7.17E-12
#3	FKBP8	90	19	1.4	5.71E-17
#3	NPDC1	88	22	1.4	1.86E-16
#3	GRN	81	28	1.4	1.56E-15
#3	UQCR10	76	18	1.398	4.95E-17
#3	TCEA1	71	18	1.398	7E-13
#3	TRPA1	56	7	1.398	2.9E-11
#3	TUFM	81	18	1.397	9.58E-16
#3	MTDH	76	19	1.397	5.24E-14
#3	VPS35	88	22	1.396	1.44E-20
#3	ATP5O	83	21	1.395	8.6E-18
#3	MGAT4A	80	16	1.395	2.36E-15
#3	SRSF3	80	21	1.394	1.27E-16
#3	FCGRT	78	21	1.394	6.61E-16
#3	CCT3	83	19	1.393	1.2E-12
#3	TMEM263	53	8	1.393	3.1E-10

#3	40057	85	20	1.392	6.09E-17
#3	MYEOV2	81	17	1.392	1.87E-17
#3	UQCRB	81	18	1.391	9.84E-20
#3	CBX4	80	20	1.391	1.61E-15
#3	HIST1H2BD	66	12	1.389	2.5E-12
#3	STXBP1	75	18	1.388	5.19E-15
#3	OS9	86	21	1.387	5.03E-16
#3	ESD	69	17	1.386	3.56E-17
#3	MCOLN3	73	15	1.384	1.15E-14
#3	APLP1	78	18	1.383	7.4E-11
#3	ATF4	81	21	1.381	1.64E-15
#3	HSPD1	73	19	1.381	2.96E-13
#3	SUMO3	78	15	1.379	1.67E-14
#3	PDE2A	71	15	1.379	9.43E-16
#3	NR3C1	80	14	1.378	9.93E-13
#3	PDCD6	76	16	1.378	4.18E-14
#3	ANXA5	76	24	1.378	3.34E-10
#3	TRNP1	76	20	1.376	3.42E-15
#3	SNX3	71	15	1.374	4.81E-14
#3	SNAP25	71	16	1.374	1.4E-10
#3	CEMIP	24	2	1.374	3.27E-06
#3	CMIP	90	27	1.373	4.89E-18
#3	COPA	83	24	1.373	4.23E-13
#3	IMPDH1	76	14	1.373	1.23E-14
#3	CACYBP	68	15	1.373	1.67E-14
#3	RARRES1	37	6	1.372	0.00003
#3	SEZ6L2	76	23	1.371	4.06E-14
#3	TRAM1	83	20	1.37	1.32E-12
#3	VAMP2	80	28	1.37	5.18E-15
#3	MDH1	68	12	1.37	5.01E-13
#3	TRIM28	81	21	1.367	4.71E-18
#3	NDUFA7	81	16	1.367	2.79E-14
#3	UBE2D3	86	22	1.366	6.01E-16
#3	FOXN3	86	23	1.366	2.01E-14
#3	HMGA1	85	18	1.365	6.96E-15
#3	ATP8A1	78	15	1.365	2.41E-13
#3	IMPAD1	85	18	1.364	1.01E-14
#3	POMP	73	19	1.364	8.81E-11
#3	SPCS2	69	14	1.364	3.69E-13
#3	RYBP	81	19	1.363	7.05E-13
#3	SEC31A	73	19	1.363	6.57E-12
#3	SOD1	88	22	1.362	8E-16
#3	PHGR1	88	37	1.361	8.01E-15
#3	NUCKS1	86	26	1.361	1.16E-16
#3	AP2S1	78	17	1.361	2.79E-14
#3	EIF3E	86	20	1.36	1.46E-15
#3	TSTD1	80	14	1.36	1.15E-16
#3	NDUFA5	86	18	1.359	4.75E-13
#3	PTP4A1	81	22	1.358	5.02E-17
#3	NUCB1	81	16	1.358	3.72E-15
#3	TMEM30B	80	15	1.358	7.65E-15
#3	SEPHS2	80	19	1.358	1.82E-14
#3	NQO1	78	19	1.358	5.83E-10
#3	PAFAH1B2	86	21	1.357	1.13E-13
#3	PTPRF	83	26	1.357	3.45E-13
#3	RBX1	71	19	1.357	1.38E-16
#3	CSNK2B	81	20	1.356	1.52E-13
#3	BANF1	76	19	1.356	5.25E-13
#3	TMEM184B	85	17	1.355	8.28E-18
#3	SLIRP	81	19	1.355	1.24E-13
#3	HIPK3	63	10	1.354	1.02E-12
#3	RAN	78	19	1.353	5.39E-12
#3	NDUFA3	76	21	1.352	6.01E-16
#3	RAB10	80	19	1.351	1.17E-15
#3	RABAC1	80	18	1.35	1.29E-15
#3	YIF1A	75	13	1.349	9.77E-12
#3	PGRMC2	75	15	1.347	1.36E-14
#3	FAM3C	66	15	1.347	5.32E-11
#3	DPYSL2	76	17	1.345	2.75E-15
#3	CAT	73	13	1.345	1.26E-12
#3	ATRX	85	25	1.344	1.64E-15
#3	TMED3	75	14	1.344	1.41E-10
#3	SCAF11	68	18	1.344	2.1E-10
#3	YWHAG	90	20	1.343	1.21E-15
#3	CHMP2A	83	19	1.343	5.6E-14
#3	APPL1	75	15	1.343	7.37E-11
#3	MEST	66	12	1.343	3.31E-10
#3	BCAP31	81	20	1.342	1.12E-17
#3	SRRM2	90	26	1.341	4.26E-14
#3	TFAP2A	46	6	1.341	9.28E-11
#3	PAPOLA	78	18	1.339	1.73E-12
#3	NFU1	71	14	1.339	1.32E-11
#3	ACSL5	66	13	1.339	9.19E-10
#3	HNRNPD	81	24	1.338	2.8E-16
#3	GPS2	78	22	1.338	6.67E-15
#3	KDELR1	92	24	1.337	1.18E-16
#3	REV3L	81	21	1.337	2.55E-11
#3	LRRFIP1	81	28	1.336	4.18E-12
#3	TBC1D1	64	12	1.336	1.54E-11
#3	LARP1	88	26	1.335	1.37E-15
#3	NMD3	68	12	1.335	6E-13
#3	SHFM1	81	20	1.334	5.52E-15
#3	UBE3A	75	18	1.334	9.22E-14
#3	SLC25A4	75	16	1.333	1.84E-10
#3	MRPS6	71	14	1.333	2.12E-12
#3	HLA-B	71	23	1.333	8.2E-09
#3	RFX3	61	9	1.332	1.69E-09
#3	LIMA1	85	21	1.331	1.46E-15
#3	SCRN1	83	16	1.331	2.05E-14
#3	KIF12	86	18	1.33	1.27E-20
#3	ETNK1	81	21	1.33	5.58E-13
#3	ERRFI1	69	10	1.33	2.38E-13
#3	PSMB6	76	19	1.328	1.33E-15
#3	RXFP4	100	0	1.327	2.94E-12
#3	STAT3	81	21	1.327	4.51E-16
#3	ARPC4	80	22	1.327	2.36E-15
#3	MLXIP	71	17	1.327	6.81E-15
#3	HADHA	68	17	1.327	1.04E-10
#3	ETFB	80	19	1.326	2.87E-16
#3	ATP6AP2	78	15	1.325	2.67E-12
#3	DUSP16	73	15	1.325	2.58E-12
#3	ADIPOR1	71	16	1.325	4.08E-13
#3	HNRNPA1	80	22	1.324	1.77E-15

#3	MIEN1	76	19	1.323	6.74E-16
#3	EPB41L4B	76	11	1.323	5.45E-11
#3	EPS8L3	76	22	1.323	5.45E-09
#3	WAC-AS1	80	22	1.322	3.4E-14
#3	PARP1	83	18	1.32	3.07E-09
#3	ATP5C1	78	18	1.32	1.42E-15
#3	TGFBI	58	10	1.32	1.23E-09
#3	USP7	73	16	1.317	9.89E-13
#3	CCT4	69	16	1.317	1.53E-10
#3	EBP	76	13	1.316	3.52E-11
#3	C11ORF31	80	19	1.315	1.73E-15
#3	METTL9	69	15	1.315	4.34E-14
#3	CAPZA1	68	21	1.315	2.93E-12
#3	ARHGDIA	85	22	1.314	7.27E-16
#3	KTN1	85	24	1.314	1.84E-11
#3	KRTCAP2	81	22	1.314	6.37E-18
#3	CTBP2	86	21	1.313	1.37E-13
#3	TMED2	86	22	1.313	3.99E-13
#3	CAMLG	75	13	1.313	1.92E-12
#3	RBM39	83	23	1.312	2.31E-12
#3	CDCP1	81	19	1.312	3.25E-12
#3	AK3	81	19	1.311	1.36E-12
#3	COMT	71	14	1.311	1.04E-12
#3	PPP2R1A	78	22	1.31	2.97E-14
#3	ATP5H	73	18	1.31	1.65E-14
#3	AP1M2	76	17	1.309	9.46E-11
#3	SQLE	69	13	1.308	5.01E-13
#3	ATP6V0D1	68	18	1.308	6.45E-12
#3	NDUFB3	68	14	1.308	5.89E-10
#3	C14ORF166	76	14	1.307	8.6E-11
#3	MED13	76	19	1.305	2.2E-14
#3	RPS14	76	25	1.303	1.51E-12
#3	EPRS	73	16	1.303	7.37E-11
#3	SRSF8	75	16	1.302	1.76E-11
#3	NUCB2	64	12	1.302	9.56E-11
#3	FAM105A	64	15	1.298	9.89E-13
#3	DAD1	68	18	1.297	9.25E-14
#3	RCAN2	68	15	1.296	1.62E-13
#3	C7ORF73	75	17	1.295	2.22E-13
#3	ATP6V1F	75	18	1.294	1.77E-12
#3	MBTPS1	75	14	1.294	1.82E-11
#3	ABHD3	54	10	1.294	1.37E-10
#3	PRR13	83	19	1.293	3.18E-15
#3	CHCHD10	76	16	1.293	1.2E-13
#3	AHCYL1	88	25	1.292	6.22E-15
#3	PPP2R4	81	16	1.292	2.75E-16
#3	CCT6A	76	18	1.291	6.9E-13
#3	GNB2	90	19	1.29	4.67E-17
#3	HMGB1	88	26	1.29	2.8E-15
#3	ROCK2	78	19	1.29	2.59E-16
#3	BCLAF1	78	21	1.29	2.54E-13
#3	ATP5I	86	21	1.289	3.88E-12
#3	PHIP	78	23	1.288	3.92E-16
#3	DHCR7	69	13	1.288	2.2E-11
#3	SPINK4	49	8	1.288	6.04E-07
#3	RIMS2	78	21	1.287	8.4E-14
#3	ELOVL5	64	11	1.287	3.86E-13
#3	NCKAP1	81	17	1.286	5.15E-16
#3	PDCD6IP	80	17	1.286	6.36E-13
#3	PLS1	76	18	1.286	8.14E-13
#3	USP22	75	23	1.285	2.6E-14
#3	EIF2AK1	71	21	1.285	6.29E-13
#3	GSG1	47	8	1.285	2.3E-09
#3	LYPLA1	71	14	1.284	2.07E-10
#3	NME4	63	12	1.284	3.84E-14
#3	ADGRG1	61	8	1.284	7.41E-13
#3	NCOR1	92	23	1.283	9.14E-13
#3	NPM1	86	21	1.283	4.68E-14
#3	TEAD1	78	19	1.283	9.21E-12
#3	CNPY2	68	16	1.283	8.72E-09
#3	SERINC1	85	19	1.282	9.42E-17
#3	FAM210B	83	23	1.282	7.54E-14
#3	MAL2	71	16	1.282	5.49E-13
#3	STRAP	75	14	1.281	3.18E-14
#3	AP2B1	75	15	1.281	2.12E-12
#3	SCOC	61	15	1.281	2.64E-11
#3	NHP2L1	81	19	1.28	1.01E-11
#3	HADHB	78	15	1.28	8.98E-13
#3	TMED9	76	16	1.28	1.76E-11
#3	C2CD4B	49	10	1.28	5.55E-07
#3	LRPAP1	76	17	1.279	1.17E-15
#3	CLOCK	78	17	1.278	8.14E-13
#3	SLC7A1	64	12	1.278	3E-10
#3	SUB1	81	18	1.276	9.98E-14
#3	RBM8A	75	15	1.276	2.72E-15
#3	HUWE1	71	17	1.276	2E-11
#3	MAGED2	71	15	1.276	2.64E-10
#3	SLC5A3	66	11	1.274	6.23E-10
#3	PLCG2	63	13	1.274	1.26E-12
#3	LOC728392	75	16	1.273	6.96E-15
#3	CNBP	71	17	1.273	3.83E-11
#3	PRRC2B	83	21	1.272	5.55E-16
#3	ATP5F1	80	18	1.272	4.61E-14
#3	ZNHIT1	78	20	1.272	4.08E-13
#3	ACTR1A	75	17	1.272	6.05E-17
#3	PSME2	69	17	1.272	2.32E-11
#3	HDAC2	85	22	1.271	8.73E-15
#3	SEC23B	69	13	1.271	2.75E-10
#3	RAP1A	68	14	1.271	2.03E-10
#3	DHCR24	75	17	1.269	6.17E-14
#3	UBE2R2	78	17	1.268	5.73E-15
#3	COX14	85	13	1.267	5.2E-15
#3	OTUB1	88	23	1.266	1.37E-13
#3	NBEA	73	17	1.266	1.35E-10
#3	NR4A1	66	12	1.266	3.37E-07
#3	FAM219B	71	15	1.265	2E-11
#3	CPNE3	81	16	1.264	1.09E-12
#3	KIF1A	80	21	1.264	8.77E-14
#3	TMEM131	69	12	1.264	1.9E-11
#3	CDX2	68	19	1.264	1.13E-10
#3	FKBP2	75	21	1.263	1.64E-12
#3	COPB1	69	15	1.262	1.09E-09
#3	OCIAD2	76	20	1.26	1.11E-13

#3	MAN1A1	71	18	1.26	2.01E-14
#3	SRSF6	71	17	1.26	3.83E-11
#3	THEM6	69	16	1.26	2.35E-12
#3	CCT5	66	15	1.26	1.86E-09
#3	DNAJC22	53	11	1.26	4.94E-09
#3	TXNL4A	75	15	1.259	3.02E-13
#3	SNRNP70	75	22	1.259	2.46E-12
#3	SSR3	71	14	1.259	6.33E-12
#3	ALCAM	80	17	1.258	2.66E-13
#3	DCTN1	81	20	1.257	3.25E-13
#3	ORMDL3	76	14	1.257	1.34E-13
#3	ATP1A1	75	18	1.257	3.72E-13
#3	RAB14	83	19	1.256	2.34E-13
#3	RAB11B	78	21	1.256	2.06E-16
#3	ABCD3	75	16	1.256	5.25E-13
#3	ZMYND11	75	15	1.256	1.46E-12
#3	ATP6V0E1	76	20	1.254	4.63E-12
#3	GNS	75	20	1.254	5.88E-13
#3	PHLDA1	47	8	1.254	9.92E-07
#3	ARF5	78	18	1.253	3.51E-10
#3	FBXO9	73	19	1.253	2.77E-11
#3	C12ORF57	69	16	1.253	1.68E-10
#3	SND1	76	17	1.25	1.02E-12
#3	GSE1	73	19	1.249	2.57E-13
#3	DNAJC10	71	19	1.248	1.14E-09
#3	TMEM70	63	12	1.248	1.86E-09
#3	NENF	69	14	1.247	2.2E-11
#3	AMIGO2	66	12	1.247	4.01E-09
#3	ZMYND8	73	21	1.246	4.4E-10
#3	SDCBP	81	18	1.245	4.97E-10
#3	SNHG16	78	16	1.245	1.62E-13
#3	SF3B1	78	20	1.244	2.85E-15
#3	38777	76	20	1.244	2.78E-13
#3	MAP1B	88	27	1.243	9E-16
#3	PTBP1	81	21	1.243	6.67E-15
#3	TPM1	80	22	1.243	4.13E-17
#3	MLXIPL	78	23	1.243	4.56E-12
#3	CREB3L1	76	18	1.243	1.04E-10
#3	TMEM205	73	18	1.243	1.24E-10
#3	MGST2	61	14	1.243	2.63E-12
#3	CMAS	71	16	1.242	6.69E-13
#3	NDUFV2	71	19	1.242	4.5E-12
#3	OGT	64	12	1.242	2.72E-10
#3	ATP5D	76	17	1.239	5.8E-16
#3	GNG5	69	18	1.239	3.92E-13
#3	BMP7	64	12	1.239	1.07E-07
#3	PSMF1	75	18	1.238	5.21E-13
#3	ANXA7	73	16	1.238	2E-11
#3	HNRNPA3	78	20	1.237	8.44E-10
#3	SERINC2	73	19	1.237	5.53E-09
#3	H2AFZ	75	16	1.236	1.6E-12
#3	CNIH2	75	17	1.235	2.27E-08
#3	VAPA	73	19	1.235	2.22E-13
#3	FADS1	68	17	1.234	1.01E-11
#3	MUC13	73	23	1.233	5.36E-12
#3	MCCC2	59	12	1.233	9.12E-12
#3	ENSA	81	21	1.232	3.8E-12
#3	ARNT2	75	14	1.232	1.09E-12
#3	SF1	71	23	1.232	8.2E-12
#3	EGLN1	53	13	1.232	3.62E-08
#3	GLO1	71	14	1.231	1.2E-11
#3	CFLAR	85	27	1.23	4.13E-14
#3	TMED7	76	15	1.23	1.66E-10
#3	GCC2	64	19	1.23	2.26E-10
#3	TMEFF2	63	11	1.23	2.03E-10
#3	MORF4L2	80	20	1.229	6.82E-13
#3	DAP	71	16	1.228	1.2E-13
#3	ARFIP2	68	16	1.228	6.72E-11
#3	C5ORF24	66	17	1.228	4.45E-10
#3	SEC11A	71	16	1.227	1.14E-10
#3	BMP4	49	19	1.227	0.00002
#3	TMEM50A	73	17	1.226	2.42E-12
#3	PSMD2	81	16	1.225	1.6E-12
#3	P4HTM	68	16	1.225	3.93E-12
#3	CDK6	68	13	1.223	2.2E-11
#3	UGDH	66	15	1.223	5.35E-10
#3	MAB21L3	75	29	1.222	1.53E-06
#3	CNIH1	58	6	1.222	3.15E-09
#3	MTCH2	85	17	1.22	1.13E-13
#3	KCNJ3	73	15	1.22	6.97E-12
#3	PSMA1	71	14	1.22	7.11E-10
#3	CREBL2	68	13	1.22	1.66E-10
#3	PPP1CC	75	17	1.219	3.49E-11
#3	SAR1B	66	15	1.219	1.17E-07
#3	ANAPC5	76	18	1.218	3.26E-14
#3	TOMM6	71	18	1.218	2.58E-12
#3	NDRG1	42	13	1.218	0.00309
#3	UFC1	86	18	1.217	3.43E-12
#3	RAP1B	73	18	1.217	2.22E-13
#3	C14ORF1	69	13	1.217	5.83E-11
#3	PRPF8	69	17	1.217	1.84E-10
#3	TAX1BP1	83	23	1.216	1.24E-11
#3	KHDRBS1	78	18	1.216	4.6E-11
#3	PCSK9	61	7	1.216	4.52E-12
#3	GK5	80	21	1.215	1.47E-09
#3	MRPL33	68	16	1.215	1.36E-14
#3	TMEM98	73	13	1.214	6.33E-12
#3	SHISA5	71	17	1.214	8.39E-12
#3	CCT2	68	16	1.214	3.56E-13
#3	42248	78	17	1.213	8.38E-14
#3	WNK1	76	18	1.213	4.77E-14
#3	COMMD6	73	15	1.213	2.46E-13
#3	EZR	88	24	1.212	8.73E-15
#3	FAM162A	69	15	1.212	9.83E-12
#3	TAF7	63	16	1.212	1.63E-12
#3	DST	81	19	1.211	1.2E-11
#3	HTATIP2	69	11	1.211	6.69E-11
#3	SPG21	68	12	1.211	5.36E-13
#3	NDUFA2	71	15	1.21	1.09E-13
#3	NDUFS2	68	15	1.21	3.65E-13
#3	LAMP1	81	18	1.209	5.78E-13
#3	ST14	75	16	1.209	4.2E-11
#3	FURIN	75	13	1.209	2.81E-10

#3	ECI2	75	14	1.208	9.83E-12
#3	ROCK1	73	19	1.208	3.26E-14
#3	IFNGR2	68	13	1.208	5.86E-11
#3	HNRNPUL1	83	22	1.207	6.57E-12
#3	DARS	78	17	1.207	4.08E-10
#3	PLD3	73	16	1.207	3.56E-13
#3	EHF	71	17	1.207	2.78E-11
#3	TPP1	66	14	1.207	2.43E-11
#3	GOLGA4	78	22	1.206	1.13E-08
#3	MAPK1IP1L	73	16	1.206	4.2E-11
#3	NBEAL2	69	20	1.206	6.1E-10
#3	FHL2	64	14	1.206	4.87E-10
#3	DYNC1I2	78	21	1.205	7.53E-12
#3	CDC42SE2	68	16	1.205	3.82E-11
#3	MRPL34	68	12	1.205	4.19E-10
#3	DLGAP4	68	16	1.204	1.09E-12
#3	WASF2	78	19	1.203	5.05E-12
#3	DSG2	80	22	1.202	2.97E-15
#3	LSS	76	15	1.202	1.03E-11
#3	SF3B2	71	17	1.202	2.29E-10
#3	RBM47	81	23	1.201	2.38E-14
#3	RAB13	81	16	1.201	4.54E-10
#3	SRSF5	80	18	1.201	5.58E-15
#3	TNPO1	68	16	1.201	2E-11
#3	TMEM258	71	15	1.2	1.38E-10
#3	NDUFB4	76	19	1.199	3.55E-12
#3	ASAH1	69	16	1.199	1.11E-11
#3	TMBIM1	61	15	1.199	1.08E-11
#3	TP53I11	71	18	1.198	3.33E-11
#3	ABHD14B	69	11	1.198	1.26E-12
#3	LASP1	76	21	1.197	9.27E-11
#3	SMARCE1	71	18	1.197	1.36E-14
#3	TMBIM4	68	17	1.197	1.3E-09
#3	PBX3	66	14	1.197	6E-13
#3	SMIM24	58	11	1.197	1.34E-12
#3	ARL6IP4	69	17	1.196	6.69E-13
#3	GALNT1	66	13	1.196	4.78E-14
#3	HNRNPM	78	17	1.195	5.52E-11
#3	EWSR1	76	17	1.195	4.18E-14
#3	ATP13A3	59	14	1.195	4.02E-10
#3	FOXA2	83	22	1.194	7.6E-14
#3	SREBF1	68	14	1.194	3.65E-10
#3	HNRNPDL	66	8	1.194	1.36E-10
#3	ZFP36L2	53	14	1.194	1.73E-08
#3	PSMC4	83	17	1.193	5.74E-12
#3	ARL1	64	13	1.193	9.66E-09
#3	VIM	51	11	1.193	8.64E-06
#3	TMEM219	80	18	1.192	3.14E-10
#3	CHP1	75	19	1.192	1.83E-15
#3	DUSP5	41	7	1.192	6.04E-07
#3	RBPJ	69	17	1.191	1.92E-10
#3	C19ORF43	85	19	1.189	2.1E-15
#3	CTTN	76	16	1.189	5.63E-15
#3	ANP32B	73	15	1.189	2.63E-13
#3	REPIN1	68	14	1.189	3.5E-12
#3	SAMD5	53	11	1.189	3.3E-08
#3	DCAF7	85	22	1.188	4.44E-14
#3	SNRPD2	85	15	1.188	7.68E-12
#3	RAD21	71	17	1.187	1.4E-10
#3	DMKN	68	14	1.187	1.24E-09
#3	KLF5	66	20	1.187	2.77E-07
#3	MT-ND4	29	42	1.187	0.00013
#3	TUG1	75	17	1.186	5.64E-14
#3	C1ORF122	73	16	1.186	6.33E-12
#3	UBE2B	73	16	1.185	4.98E-12
#3	NAPA	71	19	1.185	5.09E-14
#3	UQCRH	64	10	1.185	7.84E-09
#3	EIF3K	80	16	1.184	2E-11
#3	HSD17B10	76	11	1.184	1.36E-10
#3	PPIB	61	14	1.184	1.31E-07
#3	CTGF	47	7	1.183	0.00004
#3	HNRNPH1	83	23	1.182	4.65E-12
#3	ARC	42	11	1.182	0.00002
#3	IQGAP1	71	17	1.181	1.68E-10
#3	DDB1	68	19	1.181	1.73E-09
#3	GSTP1	81	21	1.18	2.05E-11
#3	WBP2	76	16	1.18	4.2E-11
#3	DDAH2	69	14	1.18	1.41E-12
#3	ZNF395	81	15	1.179	6.97E-12
#3	BTG1	78	21	1.179	4.76E-11
#3	MRPL51	68	13	1.178	1.56E-13
#3	HP1BP3	75	23	1.177	1.07E-09
#3	XPO6	71	13	1.177	5.32E-11
#3	RAB3IP	75	19	1.175	9.98E-14
#3	TMCO1	73	16	1.175	9.32E-12
#3	GLG1	75	19	1.174	1.31E-11
#3	GRB2	73	17	1.174	5.08E-14
#3	MYL12A	76	17	1.173	6.33E-12
#3	SCAMP5	75	17	1.173	1.04E-11
#3	STEAP2	59	9	1.173	2.2E-10
#3	MT-CO3	29	36	1.172	0.00257
#3	ILF3	75	19	1.17	6.75E-17
#3	HID1	78	19	1.169	6.9E-13
#3	NBR1	64	16	1.169	4.6E-08
#3	STMN1	81	21	1.168	4.41E-12
#3	DDX21	78	16	1.168	3.56E-13
#3	GUK1	76	18	1.168	6.17E-14
#3	SLC38A1	63	16	1.168	1.69E-09
#3	MKRN1	68	20	1.167	4.53E-10
#3	GTF3A	64	11	1.167	1.1E-09
#3	NDUFB10	76	15	1.166	3.32E-10
#3	ATP8B1	71	15	1.166	8.6E-11
#3	LDHB	68	13	1.166	3.24E-13
#3	PSMA2	68	11	1.166	9.1E-11
#3	GSPT1	64	16	1.166	2.57E-09
#3	BTG3	42	7	1.166	3.91E-09
#3	PHB	80	16	1.165	3.78E-14
#3	GNA11	71	17	1.165	1.67E-14
#3	SRP72	71	18	1.165	7.89E-12
#3	DIRC2	66	8	1.165	4.26E-11
#3	PSME1	64	15	1.165	7.38E-13
#3	CDHR3	54	13	1.165	1.42E-07
#3	PPIG	78	19	1.164	4.24E-12

#3	SNHG8	71	18	1.164	2.44E-13
#3	EIF4G3	69	16	1.164	4.2E-11
#3	IRF6	68	14	1.164	6.59E-12
#3	PXN	64	16	1.163	2.61E-11
#3	RHBDD2	76	21	1.162	1.32E-12
#3	RUNX1T1	68	15	1.162	5.99E-12
#3	PPP2CA	68	16	1.162	1.11E-11
#3	STAU1	75	17	1.161	1.4E-10
#3	MRPS21	75	16	1.161	9.49E-09
#3	PSMD7	71	18	1.161	1.21E-13
#3	PPFIA1	75	17	1.158	8.87E-14
#3	PAX6	68	15	1.158	3.14E-10
#3	MRPL20	64	15	1.158	1.34E-13
#3	LDLR	59	14	1.158	6.47E-10
#3	EIF4G1	69	17	1.157	1.76E-10
#3	PRRC2C	86	30	1.156	2.59E-10
#3	DYNC1LI2	81	19	1.156	4.93E-10
#3	SRSF7	76	14	1.155	1.71E-10
#3	SRSF1	71	19	1.155	1.42E-09
#3	HYOU1	69	19	1.155	1.35E-10
#3	NT5C3A	56	8	1.155	4.58E-10
#3	PSMA3	64	13	1.154	1.51E-10
#3	MTSS1	69	13	1.153	1.73E-12
#3	STRN4	75	18	1.152	7.64E-12
#3	NARS	66	17	1.152	2.88E-09
#3	ERGIC3	64	19	1.152	8.64E-12
#3	HDAC1	78	16	1.151	2.42E-09
#3	PLEKHB2	75	19	1.151	7E-13
#3	NDUFS6	75	19	1.151	7.49E-10
#3	FAM96B	75	18	1.151	3.41E-09
#3	C15ORF48	66	10	1.151	2.21E-12
#3	CDK2AP1	61	18	1.151	6.16E-08
#3	RAMP1	53	11	1.151	4.48E-09
#3	CELF1	86	22	1.15	5.74E-13
#3	USH1C	76	19	1.15	1.7E-11
#3	CPD	73	13	1.15	3.97E-11
#3	TMEM51	68	16	1.15	9.48E-08
#3	AMER3	66	12	1.149	2.43E-08
#3	ARL6IP1	71	19	1.148	7.2E-08
#3	ANKRD11	76	24	1.147	6.21E-10
#3	ADD1	75	16	1.147	1.09E-13
#3	LAPTM4A	73	18	1.147	4.13E-12
#3	ARPC1A	71	17	1.147	9.89E-13
#3	LTA4H	64	12	1.147	2.14E-12
#3	SSFA2	68	16	1.146	1.71E-08
#3	F3	53	16	1.146	8.48E-06
#3	MT-CYB	29	42	1.146	0.00008
#3	PTTG1IP	83	20	1.145	8.44E-10
#3	COTL1	76	13	1.145	4.51E-13
#3	SRSF11	88	29	1.144	6.31E-16
#3	VOPP1	75	18	1.144	9.34E-09
#3	AKR1A1	71	18	1.144	2.2E-10
#3	CIRBP	81	23	1.143	1.87E-15
#3	PNKD	66	12	1.143	9.93E-14
#3	UGP2	59	14	1.143	6.45E-11
#3	RAD23A	83	20	1.142	2.88E-15
#3	HIST2H2BE	73	12	1.142	2.07E-10
#3	FAM174B	68	12	1.142	1.17E-12
#3	IPO7	66	14	1.142	1.99E-11
#3	NSF	63	12	1.142	4.02E-10
#3	SKAP2	71	20	1.141	6.45E-12
#3	GOLGB1	64	20	1.14	6.34E-10
#3	BEX5	49	7	1.14	1.78E-08
#3	CDK5RAP3	80	19	1.139	9.21E-12
#3	RPS21	78	26	1.139	8.32E-10
#3	ARID1A	75	18	1.139	2.96E-13
#3	IHH	63	11	1.139	1.23E-09
#3	SLC29A1	61	10	1.139	1.71E-11
#3	UBE3C	73	14	1.138	3.97E-11
#3	SEC63	63	17	1.138	3.42E-10
#3	ABCC8	56	10	1.138	5.32E-09
#3	PPP3CA	73	17	1.137	4.2E-11
#3	HN1	71	23	1.137	1.14E-09
#3	DAZAP2	73	19	1.136	2.54E-11
#3	SYAP1	71	18	1.136	7.25E-11
#3	EIF3A	80	23	1.135	4.92E-12
#3	RAB5C	71	13	1.135	5.25E-12
#3	TUBA1C	71	16	1.135	6.47E-10
#3	PLS3	68	14	1.135	3.31E-10
#3	UBE2J1	58	15	1.135	7.82E-11
#3	ANXA11	76	20	1.134	1.5E-14
#3	VPS28	71	15	1.134	2.87E-11
#3	MT-ATP6	29	43	1.134	0.00009
#3	SEC13	71	15	1.133	8.72E-09
#3	REEP3	69	15	1.133	3.82E-11
#3	FKBP3	69	14	1.133	2.68E-08
#3	ABCB10	64	14	1.133	1.43E-09
#3	SEC24D	51	11	1.133	8.81E-06
#3	AP3D1	73	19	1.132	4.67E-11
#3	NHSL1	73	14	1.132	5.89E-10
#3	DUSP4	69	17	1.132	9.49E-11
#3	XRN2	58	12	1.132	1.23E-10
#3	GCNT1	66	15	1.131	5.23E-12
#3	MIR6087	36	6	1.131	0.00091
#3	PPP1R15B	76	12	1.13	2.02E-09
#3	TMEM238	66	14	1.13	2.37E-12
#3	YPEL5	66	16	1.13	7.35E-09
#3	XPO1	66	12	1.129	4.06E-09
#3	TPM3	85	24	1.128	1.23E-11
#3	FLNB	75	18	1.128	2.37E-12
#3	ZNF24	69	16	1.128	1.77E-12
#3	HERPUD1	69	15	1.128	2.37E-09
#3	PLXNB2	76	21	1.126	6.12E-11
#3	GFPT1	64	18	1.126	5.39E-12
#3	APOL6	69	17	1.125	9.32E-12
#3	LAMTOR4	80	14	1.124	1.26E-12
#3	TERF2IP	71	18	1.124	7.29E-09
#3	RRBP1	64	17	1.124	8.04E-08
#3	EIF1AX	85	21	1.123	6.62E-10
#3	KLHDC10	68	12	1.122	1.51E-10
#3	FAM102A	66	13	1.122	3E-10
#3	ZNF770	64	14	1.122	1.56E-12
#3	LTBR	61	12	1.122	9.77E-12

#3	CYC1	75	14	1.121	4.42E-10
#3	ACAA2	69	15	1.121	4.97E-10
#3	SLC16A3	56	10	1.121	1.09E-08
#3	TBCB	83	17	1.12	3.83E-11
#3	BAG6	75	18	1.12	5.2E-11
#3	NECAP1	71	11	1.12	1.26E-09
#3	USP9X	69	17	1.12	2.67E-07
#3	TCTA	66	11	1.12	1.39E-09
#3	ZNF638	63	17	1.12	0.00001
#3	ADCY2	59	10	1.12	4.13E-10
#3	ETFA	69	15	1.119	4.77E-09
#3	ANP32A	68	15	1.119	8.6E-11
#3	RNF11	63	14	1.119	4.4E-10
#3	CTNNBIP1	58	12	1.119	2.59E-11
#3	MARK2	76	16	1.118	4.32E-13
#3	ADAR	75	17	1.118	1.8E-12
#3	NDUFS3	63	12	1.118	1.01E-09
#3	ABHD12	61	12	1.118	1.01E-10
#3	AIM1	58	10	1.118	2.61E-07
#3	TCF25	80	21	1.117	6.57E-12
#3	MVP	69	17	1.117	3.76E-12
#3	ATN1	68	17	1.117	7.38E-13
#3	ELOVL1	66	10	1.117	5.88E-11
#3	F11R	78	20	1.116	3.02E-11
#3	TALDO1	68	15	1.116	8.47E-15
#3	ARCN1	68	19	1.115	1.31E-08
#3	TSC22D1	66	15	1.115	6.97E-12
#3	LCORL	63	13	1.114	1.2E-13
#3	C2CD4A	27	5	1.114	0.00007
#3	NDUFA4	66	19	1.113	2.29E-10
#3	KDM5B	66	15	1.113	4.01E-10
#3	HMGN2	59	11	1.113	4.41E-09
#3	FAM167A	56	8	1.113	4.25E-09
#3	PSMB2	80	17	1.112	5.52E-11
#3	AZIN1	64	12	1.112	6.86E-13
#3	PPP1R14C	63	8	1.112	1.98E-08
#3	MYO5A	54	7	1.112	3.3E-08
#3	CXXC4	86	20	1.111	4.82E-13
#3	GTF2A2	69	14	1.11	1.79E-09
#3	PIGS	68	11	1.11	3.23E-14
#3	AIDA	66	15	1.11	1.17E-07
#3	PRKCD	64	14	1.11	3.32E-10
#3	PGAM1	63	10	1.11	3.35E-10
#3	COPG1	75	17	1.109	1.34E-11
#3	OC10013125	75	32	1.109	7.03E-07
#3	SMIM15	63	16	1.109	2.88E-09
#3	ATP2C1	63	11	1.108	9.76E-08
#3	WSB1	86	27	1.106	1.31E-11
#3	SOX4	85	31	1.105	4.78E-16
#3	VDAC2	78	17	1.105	4.2E-11
#3	DDR1	71	13	1.105	5.08E-11
#3	CASK	71	11	1.105	4.06E-09
#3	GRSF1	69	16	1.105	6.75E-09
#3	UBE2H	68	18	1.105	1.25E-10
#3	COL16A1	36	6	1.105	0.00001
#3	ZC3HAV1	64	15	1.104	2.51E-07
#3	CCDC47	63	13	1.104	3.9E-11
#3	NDUFB6	69	13	1.103	7.42E-11
#3	LRP11	54	11	1.103	1.01E-11
#3	SMAD2	68	12	1.102	2.59E-11
#3	YME1L1	69	16	1.101	8.98E-13
#3	U2AF2	69	13	1.101	1.11E-10
#3	DNAJA1	69	12	1.101	2.6E-09
#3	SORT1	68	14	1.101	1.58E-08
#3	UBAP2L	66	15	1.101	1.03E-09
#3	COPS6	61	15	1.101	1.77E-11
#3	SCAND1	69	14	1.1	3.1E-12
#3	TSPYL2	56	11	1.1	2.74E-09
#3	ERH	71	16	1.099	7.82E-11
#3	EIF3F	69	13	1.099	2.37E-13
#3	MAP1LC3B	66	16	1.099	3.67E-09
#3	EMC4	68	10	1.098	8.65E-09
#3	UBE2V2	66	10	1.098	1.81E-12
#3	PELI3	66	12	1.098	1.11E-09
#3	GAK	64	15	1.098	1.8E-11
#3	NEDD8	64	15	1.098	3.03E-10
#3	STK24	59	18	1.098	4.45E-10
#3	CTDNEP1	78	18	1.097	9.31E-13
#3	ZC3H14	68	12	1.097	7.99E-09
#3	PSMA5	66	18	1.097	2.8E-09
#3	CYP27A1	69	15	1.096	1.21E-12
#3	TXNL1	61	13	1.096	3.26E-11
#3	PTGR1	59	11	1.096	1.03E-06
#3	PSMA6	73	13	1.094	7.11E-11
#3	UBE2K	68	16	1.094	2.81E-09
#3	SMARCA1	63	15	1.094	3.67E-09
#3	FBXO7	61	12	1.094	2.16E-09
#3	OCLN	53	15	1.094	9.43E-07
#3	U2AF1	71	20	1.093	1.35E-09
#3	H2AFV	73	15	1.092	8.01E-14
#3	HES6	69	17	1.092	1.36E-10
#3	TAF10	66	18	1.091	2.63E-10
#3	UQCRC2	66	18	1.091	6.86E-10
#3	ARHGEF2	61	16	1.091	3.36E-09
#3	SSTR2	59	13	1.091	2.91E-06
#3	POLR2E	69	17	1.09	8.6E-11
#3	C16ORF58	64	13	1.09	3.57E-08
#3	SFPQ	73	19	1.089	4.16E-10
#3	HSPB1	66	22	1.089	0.00002
#3	MID1IP1	54	7	1.089	1.09E-09
#3	SARS	63	16	1.088	1.42E-08
#3	MSRA	53	8	1.088	2.86E-07
#3	KCTD13	49	8	1.088	7.38E-07
#3	CAPN2	76	18	1.087	1.32E-12
#3	COPZ1	75	19	1.087	4.98E-12
#3	PNRC1	71	16	1.087	2.41E-13
#3	PSMA4	75	18	1.086	6.62E-11
#3	RPH3AL	69	15	1.086	1.99E-12
#3	TMEM147	68	12	1.086	3.1E-12
#3	ANAPC11	73	15	1.085	1.64E-09
#3	CLPTM1	69	15	1.085	2.6E-09
#3	ATRAID	68	14	1.085	5.89E-10
#3	LIN7C	68	15	1.085	9.42E-10

#3	CXCL16	54	15	1.085	2.06E-08
#3	KCNQ1OT1	90	38	1.084	1.07E-11
#3	TUBB	85	22	1.083	3.48E-12
#3	PCNP	71	14	1.083	1.55E-10
#3	TRIP12	64	15	1.083	4.95E-08
#3	ZBTB20	63	11	1.083	9.24E-09
#3	NDUFV3	75	11	1.082	4.81E-13
#3	APOOL	73	15	1.082	1.26E-10
#3	GORASP2	69	14	1.082	4.77E-11
#3	SH3KBP1	68	15	1.082	2.64E-10
#3	FAM214A	68	13	1.082	3.32E-10
#3	PPARA	63	16	1.082	3.18E-11
#3	PAFAH1B1	63	13	1.082	1.73E-07
#3	MT-CO2	29	39	1.082	0.00007
#3	UBQLN1	75	14	1.081	3.47E-15
#3	APOA1BP	75	16	1.081	1.26E-10
#3	MRPL41	69	16	1.081	2.84E-12
#3	LSM14A	66	14	1.081	1.2E-11
#3	CAPN1	66	13	1.081	2.72E-10
#3	LIPA	63	11	1.081	9.1E-11
#3	EMC7	63	12	1.081	2.26E-09
#3	TFG	59	15	1.081	3.36E-09
#3	PGD	54	13	1.081	1.23E-09
#3	STT3B	47	9	1.081	1.55E-08
#3	BPTF	75	21	1.08	2.09E-10
#3	TLN1	68	18	1.08	2.2E-10
#3	OLA1	68	16	1.08	9.42E-10
#3	MBOAT7	61	15	1.08	1.63E-09
#3	PIP5K1B	59	11	1.08	2.45E-10
#3	TM7SF3	59	14	1.08	1.86E-09
#3	PNISR	86	27	1.079	1.37E-09
#3	H2AFJ	64	11	1.079	2.24E-09
#3	RNF7	71	15	1.078	9.42E-10
#3	ANG	59	9	1.078	2.72E-10
#3	WIPF2	75	17	1.077	6.48E-10
#3	NRD1	63	15	1.077	2.81E-09
#3	BAX	58	8	1.077	6.33E-10
#3	TDG	76	18	1.076	5.68E-09
#3	SERINC3	68	16	1.076	7.37E-11
#3	MT-CO1	29	40	1.076	0.00011
#3	BRI3	71	19	1.075	6.02E-12
#3	RBMX	71	15	1.075	1.32E-11
#3	LDLRAP1	61	11	1.075	3.8E-10
#3	TMEM53	61	13	1.075	4.47E-07
#3	POU2F2	44	7	1.075	0.00007
#3	39326	69	14	1.074	3.76E-09
#3	C1ORF210	61	11	1.074	6.23E-10
#3	NPC2	71	16	1.073	8.39E-12
#3	POLR2K	64	14	1.073	1.32E-11
#3	BIVM	68	17	1.072	2.63E-10
#3	FOXP4	66	15	1.072	3.31E-08
#3	IER3IP1	64	12	1.072	6.59E-12
#3	CHD4	86	24	1.071	1.75E-10
#3	PRPF40A	69	20	1.071	3.47E-09
#3	TACC1	68	14	1.071	9.56E-11
#3	EIF4EBP2	61	15	1.071	1.73E-12
#3	SNRNP200	78	12	1.07	7.42E-11
#3	ACTN1	80	23	1.069	1.49E-07
#3	SDF4	66	13	1.069	1.55E-10
#3	DDAH1	68	17	1.068	2.19E-10
#3	SDHB	64	14	1.068	2.68E-11
#3	TTC37	59	15	1.068	2.51E-07
#3	NDUFB7	69	17	1.067	2.18E-08
#3	ITPR3	68	14	1.067	3.5E-07
#3	NHP2	63	11	1.067	6.69E-11
#3	SLC25A33	61	9	1.067	2.3E-09
#3	TRMT112	58	15	1.067	2.81E-09
#3	ACBD3	68	16	1.066	1.24E-09
#3	PPP2R5C	63	12	1.066	3.43E-10
#3	RABGAP1L	71	17	1.065	1.79E-13
#3	FAHD1	66	13	1.065	1.05E-10
#3	TERF1	63	14	1.064	2.85E-09
#3	IBTK	51	11	1.064	0.00013
#3	ATP6V0A1	66	14	1.063	8.22E-11
#3	REEP4	61	12	1.063	1.28E-08
#3	SPOP	71	16	1.062	2.12E-07
#3	ABHD16A	68	15	1.062	2.15E-11
#3	ITPK1	68	15	1.062	2.37E-11
#3	LYSMD2	63	11	1.062	1.13E-11
#3	C4ORF3	53	13	1.062	4.65E-07
#3	TRIM27	63	13	1.061	4.16E-08
#3	TIMM17B	69	12	1.06	1.41E-08
#3	MPZL1	64	13	1.06	6.69E-11
#3	WDR83OS	63	14	1.06	2.6E-09
#3	TBC1D9B	63	12	1.06	6.6E-09
#3	GLTPD2	54	5	1.06	2.57E-10
#3	VIL1	64	15	1.059	8.58E-10
#3	PUM1	75	21	1.058	1.09E-08
#3	PLEKHA1	69	15	1.058	4.01E-09
#3	GPAA1	66	14	1.058	8.66E-11
#3	CISD2	63	12	1.058	1.99E-08
#3	TMEM208	61	9	1.058	7.31E-08
#3	REPS2	54	7	1.058	2.2E-10
#3	DENR	71	16	1.057	5.08E-11
#3	WDR34	64	11	1.057	3.3E-08
#3	MRPL3	61	10	1.057	2.54E-10
#3	ATXN10	61	13	1.057	3.32E-09
#3	UGGT1	56	12	1.057	1.46E-06
#3	GLIS3	47	7	1.057	1.4E-07
#3	RAB11FIP1	73	21	1.056	4.4E-10
#3	POLR1D	64	16	1.056	1.14E-10
#3	MCF2L	66	15	1.055	1.82E-10
#3	CELF3	61	12	1.055	3.31E-10
#3	ZFR	61	15	1.055	9.24E-09
#3	CHD9	78	21	1.054	3.19E-12
#3	GAS5	78	13	1.054	5.11E-10
#3	KRT10	73	11	1.054	3.01E-08
#3	TRAPPC1	66	15	1.054	1.79E-09
#3	USP34	59	17	1.054	1.53E-10
#3	TJP2	71	12	1.053	5.08E-10
#3	GTF3C6	69	15	1.053	1.04E-10
#3	STX3	73	15	1.052	1.53E-10
#3	WBSCR22	69	17	1.052	1.4E-10

#3	BRD2	66	21	1.052	2.53E-09
#3	FBXW5	64	12	1.052	1.62E-12
#3	CERS6	63	11	1.052	2.9E-11
#3	OPTN	66	17	1.051	4.4E-10
#3	LMBRD1	63	8	1.051	6.48E-09
#3	GTF3C1	61	12	1.049	5.65E-10
#3	MAN2A1	54	11	1.049	7.31E-08
#3	NUDC	66	19	1.048	1.24E-10
#3	ATP6V1E1	63	14	1.048	3.03E-10
#3	CFAP36	42	7	1.048	3.8E-10
#3	BACE1	64	16	1.047	9.49E-09
#3	GDAP1	64	15	1.047	7.23E-07
#3	BBX	53	14	1.047	1.07E-07
#3	UBR4	69	16	1.046	1.07E-06
#3	DPY30	61	11	1.046	3.34E-09
#3	SNRPB	73	14	1.045	2.15E-09
#3	PET100	69	17	1.045	7.35E-09
#3	CAPRIN1	76	18	1.044	1.47E-09
#3	AP1G1	66	15	1.044	3.03E-10
#3	LAMTOR5	66	17	1.044	6.27E-08
#3	KIAA2013	64	14	1.044	4.41E-09
#3	ENO2	69	16	1.043	8.72E-09
#3	NGRN	68	13	1.043	1.99E-11
#3	PTPRA	64	12	1.043	2.74E-09
#3	DDX1	56	15	1.043	4.48E-08
#3	PURA	76	17	1.042	4.99E-08
#3	GNAO1	71	16	1.042	2.19E-11
#3	VEGFB	64	13	1.042	8.35E-10
#3	YTHDF2	75	19	1.041	1.56E-11
#3	GOLPH3	63	15	1.041	1.84E-10
#3	SZRD1	63	14	1.04	8.58E-10
#3	GLYCTK	59	10	1.04	1.63E-08
#3	MRPL57	49	7	1.04	1.1E-09
#3	EXOC7	68	16	1.039	2.57E-09
#3	TMEM123	61	14	1.039	4.51E-09
#3	PTPN11	59	13	1.039	2.43E-08
#3	NSA2	73	15	1.037	1.5E-11
#3	HM13	71	14	1.037	1.49E-09
#3	CS	68	14	1.037	1.97E-09
#3	DNAJB6	66	18	1.037	2.96E-10
#3	TMED4	59	9	1.037	1.03E-09
#3	SLC22A18	53	11	1.037	3.26E-07
#3	LRRC42	63	11	1.036	7.26E-09
#3	ROR1	53	9	1.036	4.18E-07
#3	IDH3G	71	11	1.035	2.48E-09
#3	SEC16A	69	15	1.035	3.32E-10
#3	NAA38	54	11	1.035	1.6E-10
#3	TSPYL1	66	14	1.034	1.45E-08
#3	CDIPT	64	15	1.034	1.66E-10
#3	PHB2	63	15	1.034	3.03E-10
#3	MRPS14	63	10	1.034	8.88E-08
#3	ARHGAP1	75	16	1.033	1.2E-09
#3	TMEM8A	58	13	1.033	1.23E-09
#3	TBL1XR1	66	15	1.032	4.82E-10
#3	SNRPG	64	14	1.032	5.89E-10
#3	CHDH	63	12	1.032	1.51E-10
#3	PDHB	63	13	1.032	1.4E-08
#3	TXNDC17	59	12	1.032	4.48E-09
#3	WASF3	54	5	1.032	1.31E-08
#3	HNRNPUL2	73	14	1.031	5.08E-10
#3	FAM60A	66	18	1.031	3.11E-07
#3	DCAF10	61	15	1.031	1.26E-10
#3	ITPR2	58	11	1.031	5.44E-09
#3	SDC4	64	15	1.03	3.16E-11
#3	PDX1	56	15	1.029	2E-08
#3	PPP3CB	54	11	1.029	5.99E-09
#3	PSMD11	78	18	1.028	9.61E-11
#3	LAMTOR2	59	14	1.028	2.37E-09
#3	PKIB	54	12	1.028	2.43E-08
#3	KRT7	75	24	1.027	3.64E-11
#3	ARRDC4	63	13	1.027	6.6E-09
#3	LRPPRC	61	11	1.027	2.07E-07
#3	PCMTD1	63	17	1.026	1.33E-08
#3	COPS5	61	10	1.026	2.42E-08
#3	RNF114	64	12	1.025	7.11E-10
#3	XIAP	63	17	1.025	1.65E-08
#3	AHI1	59	15	1.025	3.94E-07
#3	TCEB1	56	11	1.025	7.84E-09
#3	GOLGA5	54	12	1.025	7.62E-08
#3	HEBP2	73	18	1.024	9.22E-12
#3	RAB18	59	12	1.024	2.07E-08
#3	BCKDHB	56	10	1.024	1.88E-06
#3	SMARCA5	73	16	1.023	1.25E-06
#3	C4ORF48	71	16	1.023	2.64E-10
#3	ACTR3	61	13	1.023	1.45E-08
#3	GLTSCR2	73	17	1.022	3.16E-10
#3	TMEM230	71	17	1.022	6.36E-13
#3	TARS	66	13	1.022	7.02E-09
#3	MGAT5	64	13	1.022	8.94E-08
#3	HAGH	63	11	1.022	5.45E-11
#3	SHROOM3	76	20	1.021	2.25E-08
#3	LSM4	68	15	1.021	2.15E-11
#3	ARF6	68	16	1.021	9.24E-09
#3	TPST2	58	12	1.021	1.17E-08
#3	UBE2A	53	11	1.021	1.88E-08
#3	RBM17	68	17	1.02	8.01E-09
#3	SMAP1	63	12	1.02	2.07E-08
#3	CAPZB	76	19	1.019	4.5E-12
#3	CALCOCO2	68	16	1.019	9.22E-10
#3	VEZF1	64	15	1.019	6.35E-10
#3	SESN2	61	12	1.019	5.99E-09
#3	PFKL	58	13	1.019	7.26E-09
#3	RABL6	63	18	1.018	5.37E-10
#3	SMEK2	61	12	1.018	3.97E-08
#3	SUCO	58	13	1.018	2.92E-07
#3	DEGS2	53	8	1.018	5E-12
#3	TOX3	66	17	1.017	4.55E-07
#3	TRAK1	66	16	1.016	7.46E-08
#3	RAB3GAP2	63	12	1.016	6.05E-07
#3	TMEM167A	63	17	1.016	9.9E-07
#3	ZDHHC20	59	12	1.016	4.94E-09
#3	MAP2K1	56	12	1.016	5.44E-09
#3	PNN	56	18	1.016	9.53E-07

#3	NIPBL	73	17	1.015	4.98E-07
#3	SLC18B1	68	12	1.015	3E-10
#3	KCNH2	66	12	1.015	9.1E-11
#3	SUPT5H	71	17	1.014	4.6E-08
#3	TTC39A	66	14	1.014	2.72E-10
#3	MFSD10	66	12	1.014	5.72E-08
#3	PBX1	68	19	1.013	5.18E-11
#3	TRA2B	71	14	1.012	1.58E-08
#3	NDUFC1	68	17	1.012	5.39E-07
#3	VBP1	53	10	1.012	8.85E-09
#3	BAZ2A	71	18	1.011	3.35E-08
#3	TUBA4A	66	14	1.011	6.46E-09
#3	IP6K2	63	15	1.011	3.16E-11
#3	MKKS	59	10	1.01	3.15E-09
#3	TRIM44	58	14	1.01	1.36E-10
#3	FAM213A	76	20	1.009	1.05E-11
#3	C7ORF50	69	10	1.009	1.29E-10
#3	CACUL1	66	14	1.009	3.8E-08
#3	UBE2D2	64	16	1.009	8.35E-10
#3	PIN1	59	11	1.009	3.72E-10
#3	NR2F6	71	14	1.008	4.16E-08
#3	C6ORF62	69	16	1.008	3.41E-09
#3	DCXR	63	12	1.008	7.63E-10
#3	PDAP1	66	18	1.007	8.01E-09
#3	KIDINS220	64	16	1.007	1.22E-08
#3	CASP2	51	12	1.007	9.19E-10
#3	IARS2	51	9	1.007	3.65E-08
#3	LCOR	73	18	1.006	2.62E-11
#3	TSG101	66	12	1.006	1.86E-09
#3	HIP1R	54	13	1.006	1.17E-08
#3	AOC1	42	6	1.006	2.19E-08
#3	SNRPD3	68	14	1.005	2.72E-10
#3	KIF13B	64	14	1.005	7.11E-10
#3	GULP1	63	10	1.005	6.82E-06
#3	KIAA0226	61	12	1.005	3.8E-10
#3	RHOBTB3	68	20	1.004	4.76E-11
#3	NPEPPS	66	15	1.003	5.32E-09
#3	ARID4B	59	15	1.003	4.37E-09
#3	SH3BP4	66	15	1.002	3.47E-08
#3	MAVS	64	15	1.002	5.68E-09
#3	MTCH1	63	15	1.002	2.19E-10
#3	RNF10	63	15	1.002	1.58E-08
#3	TRIM2	76	18	1	7.47E-10
#3	SST	69	47	-2.203	0.01214
